# The mTORC1/4E-BP1 axis represents a critical signaling node during fibrogenesis

**DOI:** 10.1038/s41467-018-07858-8

**Published:** 2019-01-02

**Authors:** Hannah V. Woodcock, Jessica D. Eley, Delphine Guillotin, Manuela Platé, Carmel B. Nanthakumar, Matteo Martufi, Simon Peace, Gerard Joberty, Daniel Poeckel, Robert B. Good, Adam R. Taylor, Nico Zinn, Matthew Redding, Ellen J. Forty, Robert E. Hynds, Charles Swanton, Morten Karsdal, Toby M. Maher, Giovanna Bergamini, Richard P. Marshall, Andy D. Blanchard, Paul F. Mercer, Rachel C. Chambers

**Affiliations:** 1 Centre for Inflammation and Tissue Repair, UCL Respiratory, Rayne Building, University College London, London, WC1E 6JF UK; 2 Fibrosis Discovery Performance Unit, Respiratory Therapy Area, Medicines Research Centre, GlaxoSmithKline R&D, Gunnels Wood Road, Stevenage, SG1 2NY UK; 3 Target Sciences, Medicines Research Centre, GlaxoSmithKline R&D, Gunnels Wood Road, Stevenage, SG1 2NY UK; 4 Cellzome, a GSK Company, Meyershofstrasse 1, 69117 Heidelberg, Germany; 5 CRUK Lung Cancer Centre of Excellence, UCL Cancer Institute, University College London, London, WC1E 6DD UK; 6 Cancer Evolution and Genome Instability Laboratory, The Francis Crick Institute, London, NW1 1AT UK; 7 Nordic Bioscience, Herlev, 2730 Denmark; 8 Fibrosis Research Group, Inflammation, Repair & Development Section, NHLI, Imperial College, London, SW3 6LY UK

## Abstract

Myofibroblasts are the key effector cells responsible for excessive extracellular matrix deposition in multiple fibrotic conditions, including idiopathic pulmonary fibrosis (IPF). The PI3K/Akt/mTOR axis has been implicated in fibrosis, with pan-PI3K/mTOR inhibition currently under clinical evaluation in IPF. Here we demonstrate that rapamycin-insensitive mTORC1 signaling via 4E-BP1 is a critical pathway for TGF-β_1_ stimulated collagen synthesis in human lung fibroblasts, whereas canonical PI3K/Akt signaling is not required. The importance of mTORC1 signaling was confirmed by CRISPR-Cas9 gene editing in normal and IPF fibroblasts, as well as in lung cancer-associated fibroblasts, dermal fibroblasts and hepatic stellate cells. The inhibitory effect of ATP-competitive mTOR inhibition extended to other matrisome proteins implicated in the development of fibrosis and human disease relevance was demonstrated in live precision-cut IPF lung slices. Our data demonstrate that the mTORC1/4E-BP1 axis represents a critical signaling node during fibrogenesis with potential implications for the development of novel anti-fibrotic strategies.

## Abstract (web summary)

The PI3K/Akt/mTOR pathway has been previously implicated in fibrosis and a pan-PI3K/mTOR inhibitor is currently under clinical evaluation for the treatment of IPF. Here the authors show that the mTORC1/4E-BP1 axis is critical for TGF-β1-induced fibrogenesis in in vitro and ex vivo models and that canonical PI3K/Akt signalling is dispensable.

## Introduction

Fibrosis, defined as the abnormal accumulation of extracellular matrix (ECM), is a pathological feature of many chronic inflammatory and metabolic diseases and is often closely linked with organ dysfunction and, ultimately, organ failure^1,2^. The importance of the stroma in influencing cancer progression is also gaining increasing recognition^1,3^. Despite this high unmet clinical need, only two anti-fibrotic drugs, Pirfenidone/Esbriet® and Nintedanib/Ofev® have been approved to date. Moreover, these agents slow rather than halt disease progression in idiopathic pulmonary fibrosis (IPF)^4,5^, the most rapidly progressive and fatal of all fibrotic conditions. The underlying etiology of IPF remains poorly understood although current evidence suggests this condition likely arises as a result of a highly dysregulated wound healing response following chronic epithelial injury on the background of a combination of genetic predisposition and environmental factors (including cigarette smoking) and cellular senescence associated with ageing^6–8^. Highly synthetic and α-smooth muscle actin positive myofibroblasts are regarded the key effector cells of the fibrogenic response during both normal wound healing and in the context of pathological fibrosis^9^, including IPF^10–13^. The persistence of these cells, as a result of a failure in apoptosis, is felt to be a key event in the initiation and progression of fibrosis^14^. In terms of key mediators involved in promoting excessive myofibroblast differentiation and fibrogenesis, current evidence points to a key role for the pleiotropic cytokine, transforming growth factor-β (in particular the TGF-β_1_ isoform), in multiple fibrotic conditions^15^. TGF-β_1_ signals through the canonical Smad pathway and several non-canonical pathways to influence cellular function in a cell-specific and cell-context dependent manner. Therapeutic strategies aimed at targeting the dysregulated TGF-β_1_ axis in fibrosis, without compromising its critical roles in tissue and immune homeostasis, are being intensely pursued^16^.

The phosphoinositide-3-kinase (PI3K)/mechanistic target of rapamycin (mTOR) signaling pathway plays a central role in regulating a broad range of fundamental cellular processes, including metabolism, cell cycle progression, proliferation, growth, autophagy, and protein synthesis^17^. Activation of class 1 PI3K results in the production of membrane-localized phosphatidylinositol-3,4,5-trisphosphate (PIP_3_) and recruitment of Akt via its pleckstrin homology domain. mTOR functions at two distinct nodes in this signaling axis. mTOR complex 2 (mTORC2) and 3-phosphoinositide-dependent protein kinase-1 (PDK1) phosphorylate Akt at the plasma membrane to stabilize the catalytic site of Akt for maximal activation^18^. Once activated, Akt phosphorylates the TSC2 subunit of the tuberous sclerosis complex (TSC), a key control switch for mTORC1. Phosphorylation and inhibition of TSC2 lead to the accumulation of GTP-bound RAS homologue enriched in brain (Rheb) and activation of mTORC1 signaling via several downstream substrates, including p70S6K and eukaryotic translation initiation factor 4E-binding protein 1 (4E-BP1)^19^.

The PI3K/mTOR pathway has previously been implicated in influencing fibroblast proliferative responses and TGF-β_1_-induced myofibroblast differentiation and collagen production^20,21^. More recently, we provided a strong scientific rationale for progressing the potent pan-PI3 kinase/mTOR inhibitor Omipalisib (GSK2126458) as a novel anti-fibrotic agent in a proof-of-mechanism trial in IPF (https://clinicaltrials.gov/ct2/show/NCT01725139)^22^. Omipalisib displays broad target specificity and may overcome functional redundancy between PI3K isoforms and compensatory feedback loops in this pathway but on-target-toxicities associated with this class of inhibitors could be limiting^23–25^.

The mechanism by which the PI3K/mTOR pathway regulates TGF-β_1_-induced collagen synthesis is poorly understood. In this study, we characterize a unique toolbox of commercially available and proprietary pharmacological inhibitors and use these in combination with CRISPR-Cas9 gene editing and siRNA approaches to deconvolute the mechanism by which pan-PI3 kinase/mTOR inhibition blocks TGF-β_1_-induced collagen synthesis in primary human lung fibroblasts (pHLFs). We show that the potent fibrogenic effects of TGF-β_1_ are mediated via the cooperation between canonical Smad3 and rapamycin-insensitive mTORC1/4E-BP1 signaling and that the canonical PI3K–Akt axis is dispensable for this response. Furthermore, ATP-competitive mTOR inhibition halts collagen synthesis in live unmanipulated precision-cut IPF lung slices. The critical role of the mTORC1/4E-BP1 axis in influencing collagen synthesis is further generalizable to fibrogenic cells implicated in the development of fibrosis in the liver and skin, as well as the stromal reaction in lung cancer. Taken together, these experiments shed light on the signaling pathways by which TGF-β_1_ exerts its potent fibrogenic effects and provide support for selectively targeting mTORC1 signaling in IPF and potentially other fibrotic conditions.

## Results

### TGF-β_1_-induced PI3K and mTOR signaling in lung fibroblasts

**Fig. 1:**
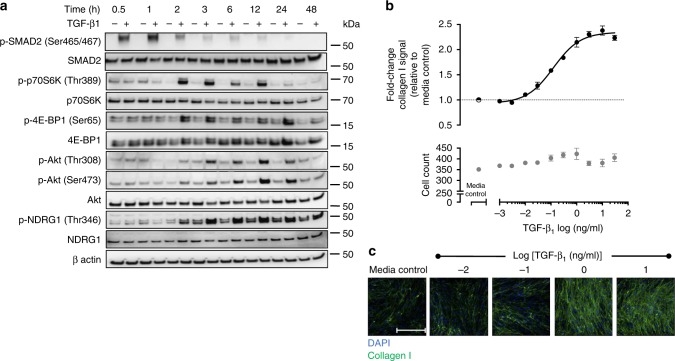
TGF-β_1_ induces Smad phosphorylation and mTOR signaling and upregulates collagen I deposition in control primary human lung fibroblasts. Control primary human lung fibroblasts (pHLFs) were stimulated with TGF-β_1_ (1 ng/ml) for 30 min to 48 h prior to cell lysis. Phosphorylation of specified proteins is shown by Western blot (**a**). Additionally, pHLFs were stimulated with increasing concentrations of TGF-β_1_ (1 pg/ml to 30 ng/ml) for 72 h with collagen I deposition assessed by macromolecular crowding assay (**b**). Data are expressed as the fold change in collagen I signal relative to the media control (4 field of view imaged per well) and cell counts obtained by staining nuclei with DAPI. Representative images are shown in **c**. Scale bars = 360 µm. Each data point shown is mean ± SEM (*n* = 4) and is representative of 3 independent experiments

Initial studies, aimed at defining the kinetics of PI3K/mTOR and canonical Smad signaling in pHLFs during TGF-β_1_-induced collagen deposition, revealed that Smad signaling was rapid and relatively short-lived with Smad2 phosphorylation peaking within the first hour and declining after 2 h (Fig. 1a). This preceded mTORC1 signaling, as evidenced by p70S6K and 4E-BP1 phosphorylation (Ser65), which was observed from 2 h onwards and was sustained for at least 12 h. Maximal PDK1-dependent phosphorylation of Akt (Thr308) and mTORC2-dependent phosphorylation of its substrates Akt (Ser473) and SGK1 (inferred by phosphorylation of the SGK1 substrate NDRG1^26^) peaked 12 h post-stimulation.

### PI3K pathway inhibition and the TGF-β_1_ collagen response

**Fig. 2:**
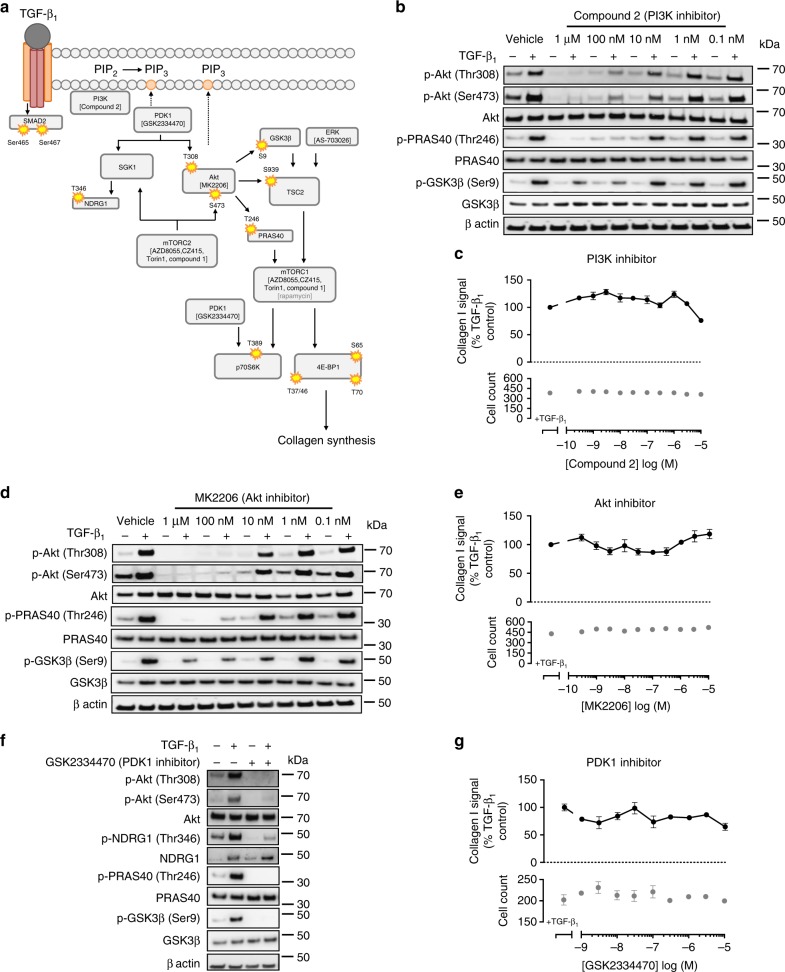
Inhibition of TGF-β_1_-induced PI3K, Akt, and PDK-1 signaling has no effect on collagen I deposition in pHLFs. A schematic, generated by author J.D.E., of inhibitors and their corresponding target is shown in **a**. pHLFs were pre-incubated with vehicle (0.1% DMSO) or increasing concentrations of Compound 2, MK2206 or 1 µM GSK2334470 prior to stimulation with TGF-β_1_ (1 ng/ml). Cells were lysed 12 h later and the phosphorylation of specified proteins was assessed by Western blot (**b**, **d**, **f**). Data are representative of 3 independent experiments. Additionally, pHLFs were pre-incubated with vehicle (0.1% DMSO) or increasing concentrations of Compound 2 (**c**), MK2206 (**e**), or GSK2334470 (**g**) and stimulated with TGF-β_1_ (1 ng/ml) for 72 h with collagen deposition assessed by macromolecular crowding assay. Data are expressed as collagen I signal calculated as a percentage of the TGF-β_1_-treated control (*n* = 4 fields of view imaged per well) and cell counts obtained by staining nuclei with DAPI. Each data point shown is mean ± SEM (*n* = 4) and is representative of 3 independent experiments

We next investigated the contribution of the PI3K/mTOR signaling axis to TGF-β_1_-induced collagen synthesis by quantitative high-content imaging of collagen I deposition under macromolecular crowding conditions^22,27^. TGF-β_1_ (1 ng/ml) increased pHLF collagen deposition by 2.1-fold (±0.1) after 72 h (Fig. 1b, c). To determine whether canonical PI3K signaling was necessary for this response, we investigated the effect of highly selective inhibitors of critical components of the PI3K/Akt axis: PI3K (Compound 2), Akt (MK2206), and PDK1 (GSK23344770) (Supplementary Fig. 1). A schematic of inhibitors used with their corresponding targets is shown in Fig. 2a. Compound 2, an ATP-competitive inhibitor selective for class 1 PI3K isoforms that exhibits low affinity for mTOR (Supplementary Table 2), attenuated both Akt Thr308 and Ser473 phosphorylation in addition to inhibiting phosphorylation of downstream substrates of Akt (PRAS40 and GSK3β; Fig. 2b). In contrast, this inhibitor had no effect on TGF-β_1_-induced collagen deposition (Fig. 2c). Similarly, treatment with either the allosteric inhibitor of Akt, MK2206, or the PDK1 inhibitor, GSK2334479, attenuated Akt phosphorylation at both Thr308 and Ser473 sites in addition to downstream Akt signaling (Fig. 2d, f) but had no effect on the TGF-β_1_-induced collagen response (Fig. 2e, g). The mTORC2 substrates, Akt and SGK1, require sequential phosphorylation by PDK1 and mTORC2 to become fully activated. TGF-β_1_-induced mTORC2-mediated downstream signaling (Akt and NDRG) was attenuated in the presence of a PDK1 inhibitor, but collagen synthesis was unaffected (Fig. 2f, g). Taken together, these data led us to conclude that canonical PI3K/Akt signaling is redundant for TGF-β_1_-induced collagen synthesis.

### The role of mTOR signaling in the TGF-β_1_ collagen response

**Fig. 3:**
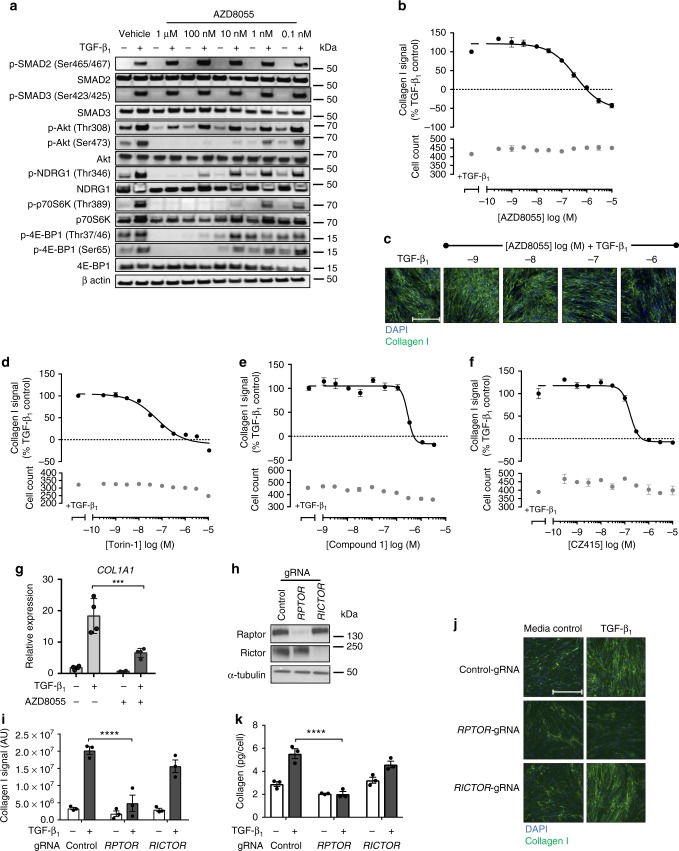
ATP-competitive inhibition of mTOR and mTORC1 knockout attenuates collagen I deposition in pHLFs. pHLFs were pre-incubated with vehicle (0.1% DMSO) or increasing concentrations of AZD8055 prior to stimulation with TGF-β_1_ (1 ng/ml). Cells were lysed at 1 h to assess Smad phosphorylation and at 12 h to assess the phosphorylation of specified proteins, assessed by Western blot (**a**). pHLFs were pre-incubated with vehicle (0.1% DMSO) or increasing concentrations of AZD8055 (**b**, **c**), Torin-1 (**d**), Compound 1 (**e**), or CZ415 (**f**) and stimulated with TGF-β_1_ for 72 h with collagen I deposition assessed by macromolecular crowding assay. Data are expressed as collagen I signal calculated as a percentage of the TGF-β_1_-treated control (*n* = 4 fields of view imaged per well) and cell counts obtained by staining nuclei with DAPI (*n* = 4). Data are representative of 5 independent experiments. Scale bars = 360 µm. IC_50_ values were calculated using 4-parameter non-linear regression: AZD8055, IC_50_ = 368 nM, 95% CI 220–616 nM; Torin-1, IC_50_ = 57.8 nM, 95% CI 38–87.7 nM; Compound 1, IC_50_ = 2.6 µM, CI 2.1–3.1 µM; CZ415, IC_50_ = 165.9 nM, 95% CI 135.4–203 nM. *COL1A1* mRNA levels were assessed by real-time RT qPCR after pre-incubation of pHLFs with vehicle (0.1% DMSO) or 1 µM AZD8055 prior to TGF-β_1_ stimulation for 24 h (*n* = 4) (**g**). Relative expression was calculated using 2^−ΔCt^. ΔCt was calculated from the geometric mean of two reference genes. pHLFs were modified by CRISPR-Cas9 gene editing using guide RNAs (gRNA) targeting exon 26 of *RPTOR* or exon 29 of *RICTOR*. Analysis of the resultant levels of Raptor and Rictor protein are shown (**h**). CRISPR-Cas9-edited pHLFs were stimulated with TGF-β_1_ (1 ng/ml) for 72 h, with collagen I deposition normalized to cell count assessed by macromolecular crowding assay (*n* = 3) (**i**). Representative images are shown in **j**. In addition, supernatants were collected from CRISPR-Cas9-edited pHLFs treated with TGF-β_1_ (1 ng/ml) for 72 h and hydroxyproline was quantified using HPLC (*n* = 3) (**k**). Data are presented as means ± SEM. Differences between groups were evaluated with two-way ANOVA with Tukey multiple comparison testing, ***p < 0.001, ****p < 0.0001

Having ruled out a role for the linear PI3K/Akt pathway, we next investigated the contribution of mTOR signaling to TGF-β_1_-induced collagen synthesis using four structurally distinct, potent and highly selective ATP-competitive mTOR kinase inhibitors that target both mTORC1 and mTORC2 (Supplementary Table 2, Supplementary Fig. 1). The first compound, AZD8055, attenuated TGF-β_1_-induced phosphorylation of the mTORC1 substrates, p70S6K (Thr389) and 4E-BP1 (Thr37/46, Ser65), as well as the mTORC2 substrates, Akt (Ser473) and SGK1 (phosphorylated NDRG1 Thr346) in a concentration-dependent manner (Fig. 3a). This compound was also highly effective at inhibiting TGF-β_1_-induced collagen synthesis (Fig. 3b, c). This observation was reproducible in fibroblasts derived from 4 additional donors (Supplementary Table 3). Similar inhibitory profiles were observed with the ATP-competitive mTOR inhibitors; Torin-1 (Fig. 3d and Supplementary Fig. 3a), Compound 1 (Fig. 3e and Supplementary Fig. 3b), and CZ415 (Fig. 3f and Supplementary Fig. 2). Supplementary Table 4 illustrates the comparative selectivity profiles of CZ415 and AZD8055 derived from the MS-based binding analysis. The complete selectivity profiles are available in Supplementary Data 1. AZD8055 also significantly attenuated the peak increase in *COL1A1* mRNA levels following TGF-β_1_ stimulation suggesting that mTOR signaling acts, at least in part, by influencing *COL1A1* mRNA levels (Fig. 3g).

Having established a key role for mTOR in mediating the fibrogenic effects of TGF-β_1_, we next investigated the relative contributions of mTORC1 and mTORC2 by CRISPR-Cas9 gene editing of either *RPTOR* (mTORC1) or *RICTOR* (mTORC2). Successful gene editing was confirmed by Western blot (Fig. 3h). TGF-β_1_-induced collagen synthesis was maintained in *RICTOR*-edited cells (Fig. 3i, j). In contrast, *RPTOR* gene editing resulted in near complete inhibition of TGF-β_1_-induced collagen synthesis. These observations were confirmed in a second assay based on the quantitation of hydroxyproline in procollagen by reverse phase HPLC (Fig. 3k) and provided strong evidence that mTORC1 is a key signaling node involved in mediating the potent fibrogenic effects of TGF-β_1_.

The PI3K pathway is considered to be the primary pathway for the activation of mTORC1 (Fig. 2a). Akt phosphorylates TSC2 at multiple sites to relieve the inhibition on Rheb and stimulate mTORC1 activity. However, neither PI3K nor Akt inhibition influenced mTORC1 downstream signaling despite attenuating TSC2 phosphorylation at the Akt-dependent Ser939 residue (Supplementary Fig. 4a, b). MAPK signaling via ERK represents a parallel pathway which also leads to the phosphorylation of TSC2^19^. Phosphorylation of TSC2 at the ERK-dependent Ser664 residue was retained when fibroblasts were stimulated with TGF-β_1_ in the presence of inhibitors targeting PI3K, Akt, or PDK1 (Supplementary Fig. 4b). In order to rule out the possibility of ERK-mediated compensation when the Akt pathway is fully inhibited, we show that the ERK inhibitor AS-703026, used either on its own or in combination with the Akt inhibitor (MK2206), did not attenuate TGF-β_1_-induced collagen deposition (Supplementary Fig. 4c, d). Inhibition of RSK1 (SL0101), which lies downstream of ERK and also phosphorylates TSC2, similarly did not inhibit the TGF-β_1_ collagen response (Supplementary Fig. 4e). This led us to conclude that TGF-β_1_-induced collagen synthesis is not dependent on Akt- or MAPK-mediated phosphorylation of TSC2.

**Fig. 4:**
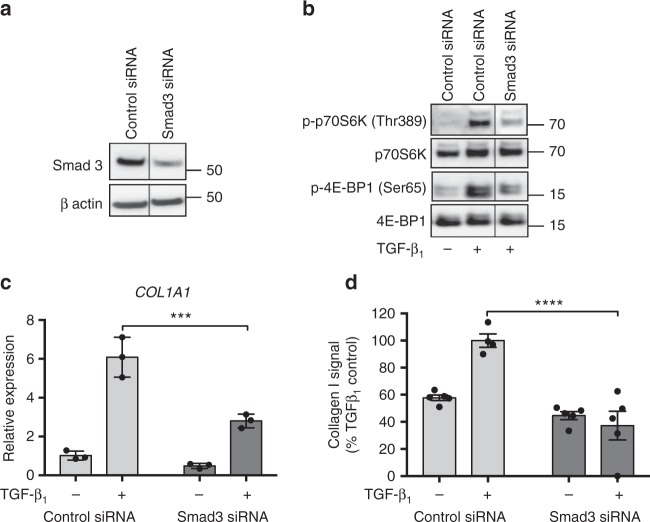
mTORC1 signaling is Smad-dependent. pHLFs were transfected with control siRNA or siRNA targeting Smad3 and Smad3 protein expression measured by Western blot (**a**). Following transfection, pHLFs were stimulated with TGF-β_1_ (1 ng/ml) for 12 h and mTORC1 signaling evaluated by Western blot analysis (**b**). An irrelevant lane has been spliced out of the prepared images (**a**) and (**b**). Uncropped gels are shown in Supplementary Fig. 9. *COL1A1* mRNA levels were assessed by real-time RT qPCR at 24 h (*n* = 3) (**c**). Relative expression was calculated using 2^−ΔCt^. ΔCt was calculated from the geometric mean of two reference genes. Collagen I deposition was measured by macromolecular crowding assay at 72 h (**d**). Data are expressed as collagen I signal normalized to cell count (*n* = 4 fields of view imaged per well) calculated as a percentage of the TGF-β_1_ -treated control (*n* = 5). Data are presented as mean ± SEM and are representative of 2 independent experiments. Differences between groups were evaluated with two-way ANOVA with Tukey multiple comparison testing, ***p < 0.001, ****p < 0.0001

Canonical Smad signaling was found to precede the activation of mTORC1 signaling in response to TGF-β_1_ stimulation (Fig. 1a), but whether Smad is necessary for the activation of mTORC1 is not known. Targeted knockdown of Smad3 in pHLFs attenuated mTORC1-dependent phosphorylation of p70S6K and 4E-BP1, indicating that the activation of mTORC1 is Smad3-dependent (Fig. 4a, b). Smad3 knockdown was also associated with a significant reduction in TGF-β_1_-induced *COL1A1* mRNA levels and collagen synthesis (Fig. 4c, d). Further support for the conclusion that Smad signaling is temporally upstream of mTOR was obtained by showing that AZD8055, Torin-1, and Compound 1 have no effect on the phosphorylation of either Smad2 or 3 (Fig. 3a and Supplementary Fig. 3).

### TGF-β_1_-induced collagen synthesis is rapamycin-insensitive

**Fig. 5:**
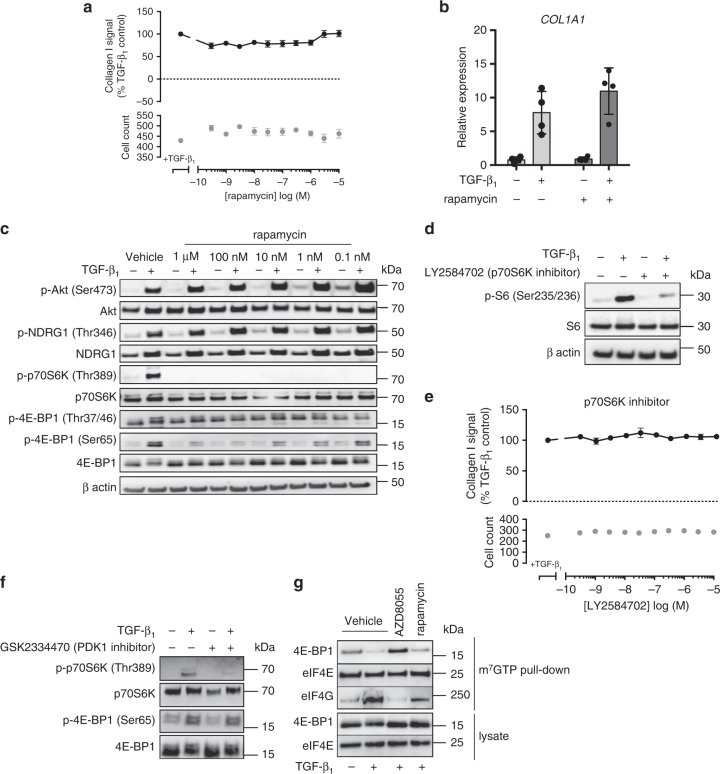
Rapamycin-insensitive mTORC1 signaling mediates TGF-β_1_-induced collagen I deposition. pHLFs were pre-incubated with vehicle (0.1% DMSO) or increasing concentrations of rapamycin (**a**) and LY2584702 (**e**) and stimulated with TGF-β_1_ for 72 h with collagen I deposition assessed by macromolecular crowding assay. Data are expressed as collagen I signal as a percentage of the TGF-β_1_-treated control (*n* = 4 fields of view imaged per well) and cell counts obtained by staining nuclei with DAPI. Data are presented as mean ± SEM (*n* = 4) and are representative of 3 independent experiments. Additionally, pHLFs were pre-incubated with vehicle (0.1% DMSO) or increasing concentrations of rapamycin (**c**), 1 µM LY2584702 (**d**), or 1 µM GSK2334470 (**f**) prior to stimulation with TGF-β_1_ (1 ng/ml). Cells were lysed 12 h later and the phosphorylation of specified proteins was assessed by Western blot. *COL1A1* mRNA levels were assessed by RT qPCR after pre-incubation of pHLFs with vehicle (0.1% DMSO) or 100 nM rapamycin prior to TGF-β_1_ stimulation for 24 h (**b**). Relative expression was calculated using 2^−ΔCt^. ΔCt was calculated from the geometric mean of two reference genes. Data are presented as mean ± SEM (*n* = 4). Differences between groups were evaluated with two-way ANOVA. For immunoprecipitation of the m^7^GTP cap, pHLFs were pre-incubated with vehicle (0.1% DMSO), 1 µM AZD8055 or 100 nM rapamycin prior to TGF-β_1_ stimulation. Protein levels were assessed by Western blot (**g**)

Having established the importance of mTORC1 signaling for TGF-β_1_-induced collagen synthesis, we examined the effect of the partial allosteric mTORC1 inhibitor, rapamycin. In contrast to ATP-competitive mTOR inhibitors, which directly bind to the mTOR catalytic site, rapamycin had no effect on TGF-β_1_-induced collagen synthesis (Fig. 5a) or the peak increase in *COL1A1* mRNA levels (Fig. 5b). Comparison of the effect of AZD8055 versus rapamycin on downstream mTORC1 substrates revealed that treatment with rapamycin completely inhibited p70S6K phosphorylation, whereas its effects on mTORC1-mediated 4E-BP1 phosphorylation were modest (Fig. 5c). In contrast, Fig. 3a shows that the ATP-competitive mTORC1/2 inhibitor, AZD8055, completely inhibited phosphorylation of both mTORC1 substrates, p70S6K and 4E-BP1, notably affecting all measured 4E-BP-1 phosphorylation sites.

To further interrogate the role of mTORC1 downstream substrates in TGF-β_1_-induced collagen synthesis, we investigated the effects of p70S6K inhibition with LY2584702. This compound (1 μM) inhibited the phosphorylation of its downstream substrate, ribosomal protein S6 (Fig. 5d), but had no effect on TGF-β_1_-induced collagen synthesis (Fig. 5e). p70S6K is a member of the AGC kinase superfamily that is sequentially phosphorylated by PDK1 and mTOR to become fully activated. PDK1 inhibition attenuated p70S6K phosphorylation by mTORC1 (Fig. 5f), but as previously mentioned had no effect on TGF-β_1_-induced collagen synthesis (Fig. 2f). Taken together, these data led us to conclude that rapamycin-sensitive mTORC1 signaling through p70S6K is dispensable for TGF-β_1_-induced collagen synthesis.

### The role of 4E-BP1 downstream of mTORC1 signaling

Having ruled out p70S6K, we next addressed the potential involvement of 4E-BP1. 4E-BP1 is a major translational repressor which inhibits cap-dependent translation by binding to eukaryotic translation initiation factor (eIF)4E, preventing the recruitment of eIF4G and the subsequent formation of the translation initiation complex. Dissociation of 4E-BP1 from eIF4E is dependent on the stepwise phosphorylation of at least four critical sites by mTORC1 (threonine residues 37, 46, and 70 and serine residue 65) and allows eIF4G to bind eIF4E to facilitate cap-dependent translation^28,29^. Cap pull-down studies with the m^7^GTP cap analog with lysates from TGF-β_1_-treated fibroblasts in the presence of either rapamycin or AZD8055 revealed that TGF-β_1_ stimulation led to the release of 4E-BP1, allowing eIF4G to bind to the cap complex (Fig. 5g). Treatment with AZD8055 enhanced the binding of 4E-BP1 to the cap complex, excluding eIF4G despite TGF-β_1_ stimulation. In contrast, rapamycin treatment failed to prevent 4E-BP1 dissociation from the cap complex in TGF-β_1_-stimulated fibroblasts. Taken together, these data suggest that only ATP-competitive mTOR inhibition, which fully suppresses 4E-BP1 phosphorylation downstream of mTORC1 to enhance 4E-BP1 binding to the cap (Fig. 3a and Supplementary Fig. 3), is capable of preventing the initiation of TGF-β_1_-induced cap-dependent translation.

**Fig. 6:**
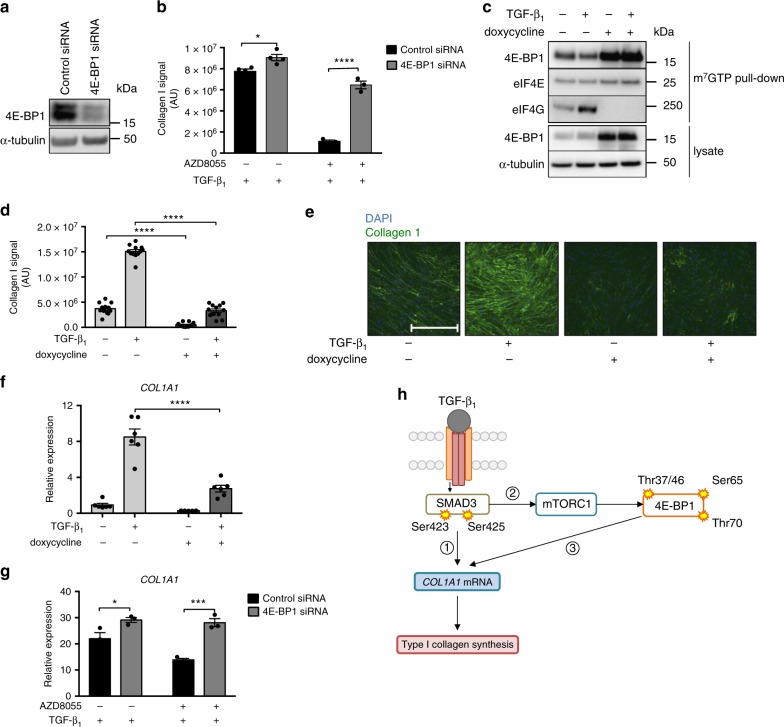
mTORC1/4E-BP1 axis mediates the TGF-β_1_ collagen I response in pHLFs. pHLFs were transfected with control siRNA or siRNA targeting 4E-BP1. 4E-BP1 protein expression was measured by Western blot (**a**). Following transfection, pHLFs were preincubated with 1 μM AZD8055 or vehicle (0.1% DMSO) prior to stimulation with TGF-β_1_ (1 ng/ml) for 72 h. Collagen I deposition was assessed by macromolecular crowding assay (*n* = 4) (**b**). *COL1A1* mRNA levels at 24 h were assessed by real-time RT qPCR (*n* = 3) (**g**). pHLFs expressing a 4E-BP1-4A dominant-negative phosphomutant were treated with doxycycline (1 μg/ml) or media control for 24 h prior to TGF-β_1_ stimulation. Immunoprecipitation of the m^7^GTP cap was performed and protein levels assessed by Western blot (**c**). Collagen deposition was measured at 72 h by macromolecular crowding assay (*n* = 12) (**d**). Representative images are shown in **e**. Scale bars = 360 µm. *COL1A1* mRNA levels were measured at 24 h *(n* = 6) (**f**). Data are presented as means ± SEM. For real-time RT-PCR, relative expression was calculated using 2^−ΔCt^. ΔCt was calculated from the geometric mean of two reference genes. For collagen deposition assays all data are expressed as collagen I signal (*n* = 4 fields of view imaged per well) normalized to cell count. All data are representative of at least 3 independent experiments. Differences between groups were evaluated with two-way ANOVA with Tukey multiple comparison testing, *p < 0.05, ***p < 0.001, ****p < 0.0001. Proposed model is shown in **h** (schematic generated by author J.D.E.). TGF-β_1_ activates the Smad3 pathway which in turn directly influences early increases in *COL1A1* mRNA levels (1), as has been previously described. Smad3 activation subsequently activates the mTORC1/4E-BP1 axis (2). This cascade regulates maximal *COL1A1* mRNA levels through an unknown mechanism, likely involving the translational regulation of a protein intermediate (3)

To further interrogate the role of 4E-BP1, we knocked down 4E-BP1 by targeted siRNA (which should mimic full 4E-BP1 phosphorylation). This led to an enhanced TGF-β_1_ response and interfered with the ability of AZD8055 to inhibit TGF-β_1_ induced collagen synthesis (Fig. 6a, b) indicating that the inhibitory effects of AZD8055 are mediated by blocking the phosphorylation of 4E-BP1. To further interrogate the mTORC1/4E-BP1 axis, we generated pHLFs expressing a doxycycline-inducible 4E-BP1 dominant negative phospho-mutant (with mTORC1 phosphorylation sites Thr37, Thr46, Ser65, and Thr70 replaced by alanine, abbreviated as 4E-BP1-4A^29^). We reasoned that the expression of 4E-BP1-4A would mimic the effects of ATP-competitive mTOR inhibition on collagen deposition. m^7^GTP cap pull-down confirmed doxycycline treatment induced 4E-BP1-4A expression and constitutive binding of 4E-BP1-4A to eIF4E (Fig. 6c). Doxycycline had no effect on 4E-BP1 expression or collagen deposition in untransduced pHLFs (Supplementary Fig. 5a, b). Expression of the dominant negative 4E-BP1-4A phosphomutant resulted in a marked attenuation of TGF-β_1_-induced collagen deposition to below constitutive baseline values (Fig. 6d, e).

4E-BP1-4A expression also inhibited TGF-β_1_-induced *COL1A1* mRNA levels (Fig. 6f); whereas 4E-BP1 siRNA knockdown similarly rescued the inhibitory effect of AZD8055 on TGF-β_1_-induced *COL1A1* mRNA levels (Fig. 6g), indicating that the mTORC1/4E-BP1 axis acts at least in part, at the level of regulating *COL1A1* mRNA levels to mediate the fibrogenic effects of TGF-β_1_ in pHLFs. Our proposed model is shown in Fig. 6h.

### mTORC1 signaling and fibrogenesis in IPF

**Fig. 7:**
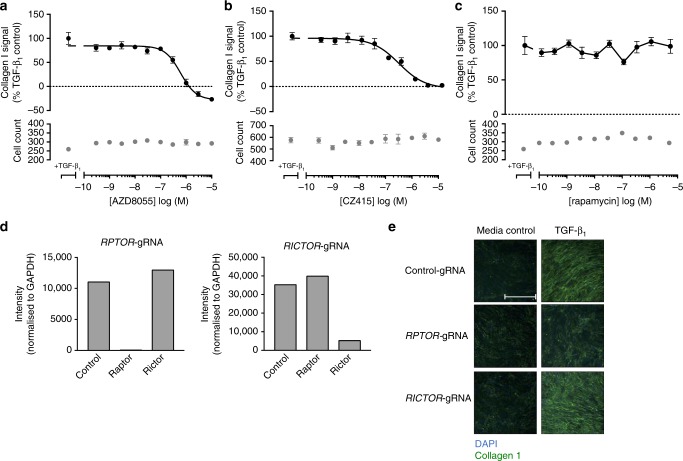
mTORC1 plays a critical role in mediating the pro-fibrotic effects of TGF-β_1_ in IPF-derived lung fibroblasts. IPF human lung fibroblasts (IPF-HLFs) were pre-incubated with vehicle (0.1% DMSO) or increasing concentrations of AZD8055 (**a**), CZ415 (**b**), or rapamycin (**c**) and stimulated with TGF-β_1_ (1 ng/ml) for 72 h with collagen I deposition assessed by macromolecular crowding assay. Data are expressed as collagen I signal calculated as a percentage of the TGF-β_1_-treated control (*n* = 4 fields of view imaged per well) and cell counts obtained by staining nuclei with DAPI. Each data point shown is mean ± SEM (*n* = 4) and is representative of 5 independent experiments. IC_50_ values were calculated using 4-parameter non-linear regression: AZD8055, IC_50_ = 604 nM, 95% CI 415 nM to 1 µM; CZ415, IC_50_ = 245.3 nM, 95% CI 136.7–541 nM. Additionally, IPF-HLFs were modified by CRISPR-Cas9 gene editing using gRNAs targeting exon 26 of *RPTOR* or exon 29 of *RICTOR*. Analysis of the resultant levels of Raptor and Rictor protein are shown (**d**). CRISPR-Cas9-edited IPF-HLFs were also stimulated with TGF-β_1_ (1 ng/ml) for 72 h, with collagen I deposition assessed by macromolecular crowding assay (**e**). Scale bars = 360 µm

All studies thus far were performed with pHLFs from control donors. Since IPF fibroblasts have been reported to be epigenetically modified (reviewed in ref. ^8^), we next examined the role of mTOR signaling in IPF-derived fibroblasts. ATP-competitive mTOR inhibition with AZD8055 (Fig. 7a) and CZ415 (Fig. 7b) was also found to inhibit TGF-β_1_-induced collagen synthesis in a concentration-dependent manner in IPF fibroblasts. This observation was confirmed in 4 additional IPF donor lines (Supplementary Table 3). As observed for control fibroblasts, the TGF-β_1_ collagen response was completely rapamycin-insensitive in IPF fibroblasts (Fig. 7c).

We were also successful in performing CRISPR-Cas9 gene editing of *RPTOR* (mTORC1) or *RICTOR* (mTORC2) in IPF fibroblasts as evidenced by marked reduction in Raptor and Rictor protein expression (Fig. 7d). As observed for control fibroblasts, TGF-β_1_-induced collagen deposition was maintained in *RICTOR*-silenced cells but *RPTOR* gene editing resulted in near-complete inhibition of TGF-β_1_-induced collagen synthesis (Fig. 7e) indicating that mTORC1 is also a key signaling node during fibrogenesis in IPF fibroblasts.

**Fig. 8:**
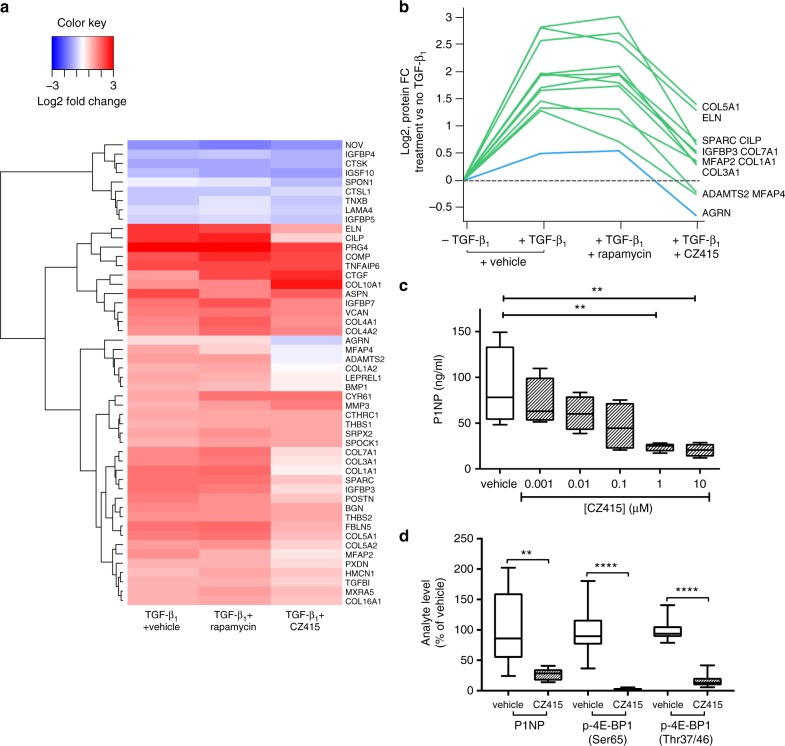
ATP-competitive mTOR inhibition attenuates TGF-β_1_-induced matrisome protein expression in vitro and collagen synthesis in IPF lung slices. IPF human lung fibroblasts (IPF-HLFs) were pre-incubated with vehicle (0.1% DMSO), rapamycin (100 nM) or the mTOR inhibitor CZ415 (5 µM) prior to stimulation with TGF-β_1_ (1 ng/ml) for 24 h. Proteomic analysis representing fold change (FC) of core matrisome proteins and proteins involved in collagen synthesis and degradation in lysates of TGF-β_1_ + vehicle/drug-treated fibroblasts versus negative TGF-β_1_ control is displayed in a heatmap. Only proteins with up- or down-regulation (*p*Adj < 0.05 and |FC| > 1.2 for both replicates) in any treatment versus negative TGF-β_1_ control are represented (50 proteins) (**a**). Quantitative profiles across treatments are displayed as line charts (**b**). Human IPF lung tissue slices generated from IPF lung transplant tissue were treated with vehicle (0.1% DMSO) or increasing concentrations of CZ415 for 120 h. Levels of P1NP in supernatants from 4 slices per condition were assessed by ELISA (**c**). Data are representative of 5 independent donors. Differences between conditions were evaluated with one-way ANOVA with Dunnett’s multiple comparison testing, **p < 0.005. Human IPF lung tissue slices (*n* = 4) from 2 donors were treated with vehicle (0.1% DMSO) or CZ415 (1 μM) for 5 days and P1NP levels in supernatants and phosphoproteins in homogenized slices were measured. Pooled donor data are presented in **d**. Differences between conditions were evaluated with Student’s *t*-test, **p < 0.005, ****p < 0.0001. In **c**, **d** the boxplot center line, bounds of box, and whiskers represent median, inter-quartile range, and minimum to maximum values

We next explored the potential role of mTOR in the broader context of regulating the TGF-β_1_-modulated matrisome by conducting a proteomic analysis of IPF fibroblasts grown under identical macromolecular crowding conditions in the presence of either CZ415 or rapamycin (Fig. 8a). We focus here on the core matrisome proteins (http://matrisomeproject.mit.edu/other-resources/human-matrisome/) and proteins involved in collagen synthesis and degradation (https://reactome.org/PathwayBrowser/#/R-HSA-1650814); the complete data is in Supplementary Data 2. Among these matrisome proteins, about 50% (169/330) were detectable in our cell system. Figure 8a represents a heatmap of the 50 proteins that showed a fold change (FC) (*p*Adj < 0.05 and |FC| > 1.2 in both replicates) in any one condition relative to the negative TGF-β_1_ control. Among these, 41 proteins showed increased levels and 9 showed decreased levels after 24 h stimulation with TGF-β_1_. To identify differences between CZ415 and rapamycin on the TGF-β_1_ response, we selected proteins that were upregulated by TGF-β_1,_ inhibited by CZ415, and insensitive to rapamycin treatment as depicted in Fig. 8b. This group included several collagen types (I, III, V, VII), as well as other ECM proteins (e.g., elastin/ELN) and matricellular proteins (e.g., SPARC) and the key procollagen processing enzyme, ADAMTS2 (also known as procollagen I N-proteinase). Of note, the procollagen processing enzyme BMP1 (responsible for cleaving the C-propeptide of fibrillar collagens) presented the same profile but just missed the significance cut-off for one replicate (*p*Adj = 0.051). Finally, the proteoglycan Agrin (AGRN) was not affected by TGF-β_1_ or rapamycin, but was significantly down-regulated by CZ415 relative to both TGF-β_1_ treatment and vehicle control levels.

In order to determine if there is a functional link between mTOR signaling and collagen synthesis in the context of human fibrotic lung disease, we next evaluated the effect of the most potent and selective ATP-competitive mTOR inhibitor, CZ415, in live unmanipulated precision-cut lung slices generated from IPF lung transplant tissue. De novo collagen synthesis was monitored by measuring the release of the human procollagen 1 formation marker; amino-terminal peptide (P1NP) by proprietary ELISA (Nordic Bioscience). Figure 8c shows that P1NP levels in slice supernatants were reduced in the presence of CZ415 in a concentration-dependent manner. This observation was confirmed in tissue slices obtained from 5 additional IPF donors (Supplementary Table 5). To confirm target engagement of the mTORC1/4E-BP1 axis, 4E-BP1 phosphorylation was measured in tissue slice homogenates following exposure to CZ415 (1 μM). Inhibition of supernatant P1NP levels was associated with significant attenuation of 4E-BP1 Ser65 and Thr37/46 phosphorylation (Fig. 8d).

### mTORC1/4E-BP1 is a core fibrogenic pathway

**Fig. 9:**
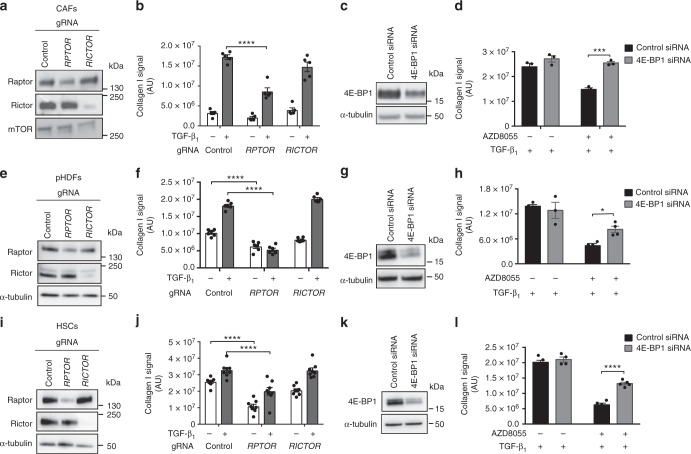
mTORC1/4E-BP1 axis mediates collagen I deposition in other mesenchymal cells. Lung adenocarcinoma-associated fibroblasts (CAFs), primary human dermal fibroblasts (pHDFs), and primary hepatic stellate cells (HSCs) were modified by CRISPR-Cas9 gene editing using gRNAs targeting exon 26 of *RPTOR* or exon 29 of *RICTOR*. Analysis of the resultant levels of Raptor and Rictor protein are shown (**a**, **e**, **i**). CRISPR-Cas9-edited CAFs, pHDFs, and HSCs were stimulated with TGF-β_1_ (1 ng/ml) for 72 h, with collagen I deposition assessed by macromolecular crowding assay (**b**, **f**, **j**). Data are expressed as collagen intensity (*n* = 4 fields of view imaged per well) normalized to cell count. Data are presented as mean ± SEM (CAFs *n* = 5, pHDFs *n* = 6, HSCs *n* = 8). CAFs, pHDFs, and HSCs were transfected with control siRNA or siRNA targeting 4E-BP and 4E-BP1 protein expression was measured (**c**, **g**, **k**). Following transfection, cells were preincubated with 1 μM (CAFs) or 300 nM (pHDFs, HSCs) AZD8055 or vehicle prior to stimulation with TGF-β_1_ for 72 h. Collagen I deposition was analyzed by macromolecular crowding assay (**d**, **h**, **l**). Data are expressed as collagen intensity (*n* = 4 fields of view imaged per well) normalized to cell count. Data are presented as mean ± SEM (CAFs *n* = 3, pHDFs *n* = 4, HSCs *n* = 5). Differences between groups were evaluated with two-way ANOVA with Tukey multiple comparison testing, *p < 0.05, ***p < 0.001, ****p < 0.0001

We also examined the relative contribution of mTORC1 and mTORC2 signaling in primary human dermal fibroblasts (pHDFs), hepatic stellate cells (HSCs), and cancer-associated fibroblasts (CAFs) derived from patients with lung adenocarcinoma by CRISPR-Cas9 gene editing to disrupt mTORC1 (*RPTOR*) and mTORC2 (*RICTOR*) signaling. These studies revealed that the magnitude of the TGF-β_1_ collagen response was variable and appeared to be related to higher baseline levels of collagen I deposition observed, especially for HSCs. Moreover, CAFs displayed evidence of heterogeneous phenotypes as has been extensively reported^30^_._ For the purpose of this study, we focused on collagen-producing CAFs. Successful gene editing was confirmed at the protein level for all lines tested (Fig. 9a, e, i). Importantly, all cell types in which *RPTOR* was disrupted were unable to mount a full TGF-β_1_ induced collagen response. In contrast, this response was fully maintained in cells in which *RICTOR* was disrupted (Fig. 9b, f, j). We also determined whether 4E-BP1 siRNA was able to restore the attenuated TGF-β_1_ induced collagen response in cells treated with AZD8055. 4E-BP1 was successfully knocked down at the protein level by targeted siRNA (Fig. 9c, g, k). In all cell types examined, 4E-BP1 siRNA rescued the impact of AZD8055 on TGF-β_1_ induced collagen synthesis (Fig. 9d, h, l), suggesting that the mTORC1/4E-BP1 axis represents a common fibrogenic signaling hub across stromal cells derived from different organs.

## Discussion

Highly synthetic myofibroblasts are the key effector cells involved in excessive ECM deposition in multiple fibrotic conditions, including IPF. We recently reported that combined pan-PI3K and mTOR inhibition with Omipalisib attenuates TGF-β_1_-induced collagen synthesis in human lung fibroblasts (HLFs)^22^. We now dissect the relative contributions of PI3K and mTOR signaling to TGF-β_1_-induced collagen deposition and report that rapamycin-insensitive mTORC1/4E-BP1 signaling is critical for this response in control and IPF-derived fibroblasts, whereas upstream canonical PI3K/Akt signaling is dispensable. Moreover, the effects of mTOR inhibition extend to other matrisome proteins implicated in the fibrotic response. Targeting mTOR by ATP-competitive mTOR inhibition further blocks collagen synthesis in live IPF tissue slices. Finally, the mTORC1/4E-BP1 axis is also critical for collagen I synthesis by dermal fibroblasts, HSCs, and lung cancer-associated fibroblasts.

PI3K/Akt signaling in response to TGF-β_1_ stimulation in HLFs has previously been implicated in pro-survival and anti-apoptotic signaling^31,32^. We now provide strong pharmacological evidence that this axis is however not required for TGF-β_1_-induced collagen synthesis in that class 1 PI3K, Akt, and PDK1 inhibitors, which robustly attenuated Akt signaling, consistently had no effect on TGF-β_1_-induced collagen deposition in our assays. Although this observation contrasts with previous studies reporting a role for PI3K in TGF-β_1_ mediated collagen synthesis^33–35^, these earlier studies employed first generation PI3K inhibitors, LY294002 and wortmannin, which inhibit a broad range of other PI3K-related proteins, including mTOR^36^. In contrast, Compound 2 used here exhibits exquisite selectivity for all class 1 PI3K isoforms over mTOR.

Whilst the PI3K/Akt axis is one of the best-characterized pathways leading to mTORC1 activation^37^, PI3K and Akt inhibition did not impact TGF-β_1_-induced mTORC1 downstream signaling. This is consistent with the temporal kinetics of the signaling response to TGF-β_1_, in that maximal phosphorylation of mTORC1 substrates was consistently observed at least 10 h before maximal phosphorylation of Akt. We considered the potential involvement of a number of potential PI3K/Akt independent pathways that could regulate mTORC1 activation in response to TGF-β_1_ stimulation. The TSC complex is a key control switch for mTORC1 activation and is phosphorylated by multiple other inputs besides Akt, including MAPK, Wingless-related integration (Wnt) signaling, redox and nutrient status^19^. Our data show that, while MAPK-dependent TSC2 phosphorylation occurs in response to TGF-β_1_, pharmacological inhibition of the major MAPKs implicated in TSC phosphorylation, ERK or RSK1, did not inhibit the downstream TGF-β_1_ collagen response. Furthermore, we ruled out the possibility of compensation between the Akt and ERK pathways, as reported in other cell contexts^38^, on the basis of a lack of an inhibitory effect observed by combined inhibition of Akt and ERK.

In contrast to the lack of evidence supporting the involvement of PI3K and Akt and MAPK signaling, ATP-competitive mTOR inhibition had a profound inhibitory effect on TGF-β_1_-induced collagen synthesis (with the most potent and selective compounds tested displaying an IC_50_ within the nanomolar range) in control and IPF fibroblasts. We subsequently established a critical role for rapamycin-insensitive mTORC1 signaling in mediating the fibrogenic responses of TGF-β_1_ on the basis of evidence obtained by CRISPR-Cas9 gene-editing of *RPTOR* and the lack of effect of rapamycin treatment on this response, as has been reported by others^39,40^.

Compared to ATP-competitive mTOR inhibitors, which directly bind to the mTOR catalytic site, rapamycin is an allosteric inhibitor that exclusively binds to the FKBP12/rapamycin-binding (FRB) domain of mTORC1 to restrict the access of substrates to the catalytic site. Previous phosphoproteomic studies have shown that the mTORC1 substrates, p70S6K and 4E-BP1, are differentially sensitive to rapamycin treatment, based on the avidity with which they are phosphorylated by mTORC1. p70S6K (Thr389) and 4E-BP1 (Ser65) are weakly phosphorylated by mTORC1 and readily sensitive to rapamycin treatment, whereas 4E-BP1 (Thr37/46) are avidly phosphorylated and insensitive to rapamycin treatment^41^. In contrast, all p70S6K and 4E-BP1 sites are sensitive to ATP-competitive mTOR inhibitors^41^. Several observations led us to exclude a role for mTORC1/p70S6K in mediating the TGF-β_1_ collagen response. First, TGF-β_1_-induced p70S6K phosphorylation was highly sensitive to rapamycin treatment, but rapamycin had no effect on either TGF-β_1_-induced *COL1A1* mRNA levels or collagen deposition. Second, the p70S6K specific inhibitor, LY2584702, did not inhibit the TGF-β_1_ collagen response. Finally, treatment with the PDK1 inhibitor (GSK2334470) prevented TGF-β_1_-induced p70S6K phosphorylation, but again had no effect on TGF-β_1_-induced collagen synthesis.

Our data provides strong support that mTORC1 mediates the fibrogenic effects of TGF-β_1_ via a 4E-BP1-dependent mechanism. Analysis of cap-dependent translation complex formation in response to TGF-β_1_ stimulation suggests that attenuation of 4E-BP1 (Ser65) phosphorylation alone by rapamycin is not sufficient to block mTORC1-mediated cap-dependent translation and further that the potent effect of ATP-competitive mTOR inhibition on TGF-β_1_-induced collagen synthesis is likely mediated via a marked inhibitory effect on the phosphorylation of multiple 4E-BP1 sites. Conclusive evidence for the critical involvement of 4E-BP1 was provided by data demonstrating that inducible expression of a dominant-negative 4E-BP1 phosphomutant led to avid 4E-BP1 binding to the 5′ cap and a profound inhibitory effect on the TGF-β_1_-induced collagen response. In addition, silencing 4E-BP1 by siRNA targeted knockdown (which effectively mimics 4E-BP1 phosphorylation), rescued the inhibitory effect of the mTOR inhibitor, AZD8055, on TGF-β_1_-induced collagen synthesis, indicating that AZD8055 exerts its anti-fibrotic effects via a 4E-BP1-dependent mechanism. These findings are consistent with a recent report suggesting the involvement of mTORC1/4E-BP1 in basal collagen synthesis in the context of bronchiolitis obliterans^40^. However, we should caution that our findings highlighting a critical role for mTORC1/4E-BP1 conflict with a previous study reporting a role for mTORC2 based on Western blotting for collagen following shRNA knockdown of *RICTOR*^39^. We are currently unable to explain these contrasting findings, but it is worth commenting that we employed the most selective pharmacological tools available to probe multiple kinases along the PI3K/Akt/mTOR axis in combination with a highly efficient CRISPR-Cas9 gene-editing approach and used two complementary methods for assessing collagen synthesis (macromolecular crowding assays and hydroxyproline levels). Further studies using conditional tissue-specific *RPTOR* and *RICTOR* deficient mice may help resolve the issue with respect to the relative contributions of mTORC1 versus mTORC2 signaling and fibrogenesis in an in vivo setting.

In terms of identifying the mechanism by which mTORC1/4E-BP1 influences the TGF-β_1_ collagen response, several lines of investigation suggest that this occurs, at least in part, at the level of regulating *COL1A1* mRNA levels. First, AZD8055 attenuated TGF-β_1_-induced *COL1A1* mRNA levels; second, the impact of AZD8055 on *COL1A1* mRNA levels could be rescued by targeted 4E-BP1 silencing; and third, inducible expression of a dominant-negative 4E-BP1 phosphomutant blocked the increase in TGF-β_1_-induced *COL1A1* mRNA levels. Given the key role of the canonical Smad pathway in mediating downstream TGF-β_1_ signaling, we also examined how mTORC1 signaling might intercept with the Smad pathway to regulate TGF-β_1_-induced collagen expression. We focused our functional interrogations on Smad3, which has been strongly implicated in mediating the fibrogenic effects of TGF-β_1_ in vitro and in vivo^42,43^. Our data show that Smad3 and mTORC1 signaling are both critical and cooperate to promote TGF-β_1_-induced collagen deposition. We now propose a potential model whereby canonical Smad3 signaling influences immediate-early *COL1A1* gene transcription as previously described^42^, but is also critical for the activation of the mTORC1/4E-BP1 axis following TGF-β_1_ stimulation. This axis in turn is critical for TGF-β_1_-induced collagen expression, at least in part, by influencing peak *COL1A1* mRNA levels, possibly by influencing the translation of an as yet unidentified protein intermediate (Fig. 6h).

Seminal ribosome profiling studies by the Sabatini laboratory have demonstrated that mRNAs with a terminal oligopyrimidine (TOP) motif in the 5′UTR (which includes ribosomal proteins and translation elongation factors) exhibit enhanced sensitivity to translational regulation by the mTORC1/4E-BP1 axis^29^. Sequence analysis of the 5′UTR of *COL1A1* and *COL1A2* mRNAs (RefSeq accession NM_000088, NM_000089, dbTSS) revealed that these transcripts do not satisfy the original criteria for TOP/TOP-like motifs. However, given the profound inhibitory effect observed by disrupting mTORC1/4E-BP1 signaling on TGF-β_1_-induced collagen synthesis, we propose that mTORC1/4E-BP1 might act at multiple levels of the collagen biosynthetic cascade. Further extensive studies, including ribosome profiling, will be required to pinpoint the precise mechanism by which mTORC1/4E-BP1 influences TGF-β_1_-induced collagen expression.

A number of ATP-competitive mTOR inhibitors are now reaching the clinical phase of development in other disease contexts (including equally fatal conditions such as cancer^44^), we therefore extended our analysis to determine the impact of ATP-competitive mTOR inhibition on other TGF-β_1_ regulated matrisome proteins in IPF-derived fibroblasts by comparing the effect of CZ415 (the most potent and selective ATP-competitive mTOR inhibitor tested, Supplementary Fig. 2) with rapamycin. Our proteomic studies revealed that there were major differences between the anti-fibrotic potential of these two compounds: in addition to inhibiting collagen I, CZ415 inhibited other major components of the fibrogenic niche including several other collagen types (III, V, VII), elastin (ELN), several matricellular proteins, and collagen processing enzymes. In contrast, the upregulation of these proteins was completely rapamycin-insensitive. Of the CZ415-sensitive proteins, only *ELN* was classifiable as a TOP mRNA whereas *COL5A1*, *MFAP2*, and *MFAP4* are classifiable as potentially TOP-like. The translation of these mRNAs may therefore be directly regulated by the mTORC1/4E-BP1 axis, further supporting the notion that ATP-competitive mTOR inhibition acts at multiple steps to modulate the TGF-β_1_ ECM response. We further propose that the lack of a direct anti-fibrogenic effect by rapamycin might, in part, explain the recent negative IPF clinical trial of the closely related rapalog, everolimus^45^.

Having established a basis for exploring ATP-competitive mTOR inhibition as a potential novel anti-fibrotic strategy, we focused on establishing a human-disease-based rationale for targeting this axis in the context of IPF. Support for this strategy in the most commonly used animal model of bleomycin-induced lung injury and fibrosis in mice became available during the course of this study based on the ATP-competitive mTOR inhibitor, MLN0128^39^. The bleomycin model is widely regarded to be helpful in terms of enabling mechanistic investigations relevant to fibrogenesis in an in vivo context. However, it also has well-recognized limitations in terms of recapitulating important features of the human disease and downstream clinical predictability^46^. We therefore elected to further investigate the potential of ATP-competitive mTOR inhibition in IPF by investigating the impact of such an approach on collagen synthesis in live precision-cut ex-vivo lung slices derived from transplant tissue from IPF patients. These studies revealed that the potent and selective ATP-competitive mTOR inhibitor, CZ415, was highly effective at inhibiting collagen synthesis in these IPF slices. Our data further provided evidence of target pathway engagement, as the anti-fibrotic effect of CZ415 was associated with inhibition of 4E-BP1 phosphorylation at residues demonstrated throughout our study to be critical for regulation of the collagen biosynthetic cascade. Future studies with clinically developable ATP-competitive mTOR inhibitors focused on determining their safety, efficacy, and therapeutic window in animal models of pulmonary fibrosis will be needed in order to further support the translatability of this approach for the treatment of IPF.

Excessive ECM deposition leading to architectural distortion and functional organ impairment is the hallmark of all fibrotic diseases^47^ and is also a feature of the stromal reaction in cancer^1^. Most of the work presented herein focused on the collagen response in the context of IPF, the most rapidly progressive and fatal of all fibrotic diseases. However, it is recognized that although the initial insult may be distinct in terms of promoting the development of fibrosis across different organs, there are likely to be common pro-fibrotic pathways that drive ECM deposition in different disease contexts^2^. Data obtained using CRISPR-Cas9 gene editing of *RPTOR* and *RICTOR* in combination with data obtained by 4E-BP1 siRNA rescue of the inhibitory effects of AZD8055 on the TGF-β_1_ collagen response provide strong support that our key observation regarding the critical role of the mTORC1/4E-BP1 signaling during fibrogenesis is generalizable to other key effector cells of the fibrotic response in the context of the skin (pHDFs), the liver (HSCs), and the stromal reaction in lung adenocarcinoma (CAFs).

In conclusion, we report evidence based on multiple lines of investigation that the mTORC1/4E-BP1 axis plays a critical role in mediating the fibrogenic effects of TGF-β_1_, the most potent pro-fibrotic mediator characterized to date. We propose that targeting this axis may hold broad promise as a potential anti-fibrotic strategy in the context of multiple fibrotic conditions and the stromal reaction in cancer.

## Methods

### Primary cell culture

pHLFs were grown from explant cultures of IPF or non-IPF control lung tissue. Briefly, lung parenchyma was cut into 1 mm^3^ fragments and placed on 10 cm tissue culture dishes with Dulbecco’s modified Eagle’s medium (DMEM) (ThermoFisher Scientific) supplemented with 10% (v/v) fetal calf serum (FCS) (Sigma-Aldrich), penicillin (100 U/ml), streptomycin (100 μg/ml), and 2.5 μg/ml amphotericin B (ThermoFisher Scientific). Fibroblasts developed into a near confluent monolayer of cells after 3–4 weeks. Experiments were conducted on cells between passages 2 and 8. CAF cell cultures were established through the Tracking Cancer Evolution through Therapy (TRACERx) clinical study (REC reference 13/LO/1546). CAFs were grown from explant culture of tumor tissue from patients with lung adenocarcinoma. Primary normal human adult dermal fibroblasts and human HSCs were purchased from Lonza (#CC-2511) and Zen-Bio (#HP-F-S), respectively.

To generate a HLF cell line expressing a dominant-negative mutant of 4E-BP1, fibroblasts were transduced with lentiviral tet-on plasmid. pCW57.1-4EBP1_4xAla was a gift from David Sabatini (Addgene plasmid #38240)^29^. Cells were selected with 2 μg/ml puromycin (Sigma-Aldrich) for 5 days. Expression of the 4E-BP1 mutant was induced with 1 μg/ml doxycycline treatment (Sigma-Aldrich) for 24 h.

For experiments analyzed by Western blot or real-time RT-PCR, cells were seeded into 6-well plates (Nunc™, ThermoFisher Scientific) and cultured for 48 h prior to serum starvation. Fibroblast cultures assessed for collagen I deposition were seeded into black-walled 96-well plates (Corning) for 24 h prior to low-serum (0.4%) starvation. All cell lines tested negative for mycoplasma.

### Immunoblotting

Fibroblasts were lysed in ice-cold PhosphoSafe® buffer (Merck Millipore) supplemented with protease inhibitors (cOmpleteMini™, Roche). Following SDS PAGE, gel to membrane transfer and blocking, protein phosphorylation was assessed by Western blot using commercially available antibodies (catalogue numbers and dilutions supplied in Supplementary Table 1), and visualization of membranes was performed using ImageQuant TL v8.1 software (GE Healthcare). Following stripping, membranes were blocked and re-probed for total levels of specified proteins. Uncropped Western blots for all figures are shown in Supplementary Figs. 6–15.

### Determination of type I collagen deposition

Type I collagen biosynthesis and deposition was determined in a 96-well format by a high-content immunofluorescence-based macromolecular crowding assay modified from a previously described method^27^. Briefly, fibroblasts were cultured in DMEM (0.4% FCS) in the presence of ascorbic acid and a dissolved mix of Ficoll 70 and Ficoll 400 as macromolecular crowding agents. For compound studies, fibroblasts were incubated either with vehicle (DMSO) or a specified inhibitor (Supplementary Table 2, Supplementary Fig. 1) for 1 h prior to stimulation with TGF-β_1_ (1 ng/ml) and further incubation for 72 h at which point cell monolayers were fixed for immunofluorescence. Fibroblasts were stained with a collagen I antibody (Sigma-Aldrich) and AlexaFluor488 secondary antibody (ThermoFisher Scientific), nuclei were counterstained with DAPI. Images of fibroblasts and collagen deposition were captured and the fluorescent signal quantified on the ImageXpress Micro XLS high-content imaging system at 20× magnification (Molecular Devices).

### *COL1A1* real-time quantitative PCR

Total RNA was extracted from adherent cells using RNeasy Mini kit (Qiagen) according to the manufacturer’s instructions. Real-time PCR was performed using a Mastercycler Realplex ep gradient S (Eppendorf). Primer sequences: *COL1A1* forward 5′ ATGTAGGCCACGCTGTTCTT 3′ and *COL1A1* reverse 5′ GAGAGCATGACCGATGGATT 3′. PCR amplification was carried out for 40 cycles at a melting temperature of 95 °C for 15 s and an annealing temperature of 60 °C for 1 min. A dissociation curve was analyzed for each PCR experiment to assess primer–dimer formation or contamination. Relative mRNA level quantifications of target genes were determined using the cycle threshold method with *ATP5B* and β2 microglobulin (*β2M*) as housekeeping genes (PrimerDesign Ltd. Primer sequences for housekeeping genes are proprietary, accession numbers supplied by manufacturer: NM_001686 (*ATP5B*), NM_004048 (*β2M*)), and the data were expressed as the expression relative to the housekeeping genes.

### CRISPR-Cas9 gene editing

Guide RNA (gRNA) sequences were designed using the Deskgen design platform (https://www.deskgen.com/guidebook/advanced.html). Primary cells were electroporated with a CRISPR ribonucleoprotein (RNP) complex targeting either *RICTOR* exon 29 or *RPTOR* exon 26 using the Lonza 4D Nucleofector™ system (Basel, Switzerland). MiSeq analysis was performed to genotype the loci that were targeted by the CRISPR RNP complex and showed 94% allele mutation frequency. Loss of Rictor and Raptor protein expression was confirmed using Western blotting. The gRNA sequences and primers used are given in Table 1.

**Table 1:** Guide RNAs and primer sequences used for CRISPR-Cas9 gene editing

	*RICTOR*, exon 29
gRNA	AATATCGGCTCATCAAATTGGGG
MiSeq forward primer	ACACTCTTTCCCTACACGACGctcttccgatctATGACCTACCCTCTGATGGAAAG
MiSeq reverse primer	TGACTGGAGTTCAGACGTGTGctcttccgatctTTTTTCTCTCTCAGAGATGAGGT

	*RAPTR*, exon 26
gRNA	CCGCGTCTACGACACAGAAGGATGG
MiSeq forward primer	ACACTCTTTCCCTACACGACGctcttccgatctACCCAACCAAATGGCAGTGACAC
MiSeq reverse primer	TGACTGGAGTTCAGACGTGTctcttccgatctTGCCTGTGTTTGGCTCTAGGACA

### siRNA transfection

Confluent cells were serum starved and transfected with 10–50 nM ON-TARGETplus siRNA pools targeting *EIF4EBP1* or *SMAD3* (Dharmacon) or Silencer Select negative control siRNA (ThermoFisher Scientific) using RNAiMax lipofectamine (Invitrogen) according to the manufacturer’s instructions.

### m^7^GTP immunoprecipitation

Fibroblasts were lysed in ice-cold Pierce immunoprecipitation lysis buffer (ThermoFisher Scientific) supplemented with EDTA-free protease and phosphatase inhibitors (ThermoFisher Scientific). Association of proteins with the 5′ mRNA cap was determined by first incubating lysates with m^7^GTP-bound sepharose beads (Jena Bioscience), followed by Western blot to assess the association of relevant proteins.

### Matrisome proteomics

IPF-derived lung fibroblasts were grown in macromolecular crowding conditions and treated with either vehicle (DMSO), rapamycin (100 nM), or the mTOR inhibitor CZ415 (5 µM). Following compound treatment for 3 h, TGF-β_1_ (1 ng/ml) was added for 24 h, and cells were harvested by scraping of the culture surface. PBS-washed cell pellets were then processed for LC-MS/MS analysis (please see Supplementary materials and methods for a detailed protocol). The mass spectrometry proteomics data have been deposited to the ProteomeXchange consortium via the PRIDE partner repository^48^ with dataset identifier PXD010164.

### Collagen synthesis in precision-cut lung slices

To generate precision-cut lung slices, IPF tissue was first inflated with 3% agarose dissolved in RPMI and cooled. 8 mm diameter cores were generated and 250 µm slices were produced using the Krumdieck tissue slicer (Alabama Research and Development). Slices were cultured for 24 h in DMEM (10% CO_2_/100% humidity) prior to incubation with the mTOR inhibitor, CZ415, for 120 h (media changed and CZ415 re-added at 72 h). The effect of CZ415 on levels of the collagen formation marker, P1NP, in tissue supernatant was assessed by proprietary competitive ELISA (Nordic Bioscience)^49^. Tissue slices were homogenized and analyzed for the phosphorylation of 4E-BP1 by MSD immunoassay (Meso Scale Discovery, USA).

### Quantification of hydroxyproline from cellular supernatants

Fibroblast procollagen production was assessed by high-performance liquid chromatography (HPLC) quantitation of hydroxyproline in supernatants from confluent fibroblast monolayers. Briefly, proteins were precipitated by the addition of ethanol to a final concentration of 67% (vol/vol) at 4 °C overnight and separated from free amino acids with 0.45 μm filters (Millipore). Filters with adherent proteins were hydrolyzed in hydrochloric acid (6 M) at 110 °C overnight. Hydrolysates were decolorized with charcoal, filtered through a 0.65 μm filter (Millipore) (Sigma Aldrich) and then derivatized with 7-chloro-4-nitrobenzo-2-oxa-1,3-diazole (Acros Organics, ThermoFisher Scientific) prior to reverse-phase HPLC (Agilent 1100 series, Agilent Technologies) for the isolation of hydroxyproline with an acetonitrile gradient using a LiChrosopher, 100 RP-18 column.

### Statistical analysis

Concentration–response curves, mRNA and tissue slice data figures were constructed using GraphPad Prism version 7.00. Four-parameter non-linear regression analyses were used to generate IC_50_ values from concentration–response curves. Additionally, where specified in relevant figure legends, data were analyzed by Student’s *t*-test, one-way ANOVA with Dunnett’s multiple-comparisons testing or two-way ANOVA with Tukey multiple-comparisons testing. Data were considered statistically significant at *p* < 0.05.

### Study approval

Samples of IPF lung tissue were obtained from patients undergoing lung transplant or surgical lung biopsy following informed signed consent and with research ethics committee approval (11/NE/0291—NRES Committee North East—Newcastle and North Tyneside 1, 10/H0504/9—National Institute of Health Biomedical Research Unit Advanced Disease Biobank, Royal Brompton Hospital, 10/H0720/12—London—Hampstead Research Ethics Committee and 12/EM/0058—NRES Committee East Midlands—Nottingham 2). Cancer-associated fibroblast (CAF) cell cultures were established within the Tracking Cancer Evolution through Therapy (TRACERx) clinical study (13/LO/1546—NRES London—Camden and Islington). Tissue for lung slice experiments was obtained from Asterand Europe (Royston, UK) in compliance with the UK Human Tissue Act 2004. The human biological samples were sourced ethically and their research use was in accord with the terms of the informed consents.

## Supplementary information

Supplementary Information

Description of Additional Supplementary Files

Supplementary Data 1

Supplementary Data 2

## Data Availability

The mass spectrometry proteomics data have been deposited to the ProteomeXchange consortium via the PRIDE repository, and are publicly available with the dataset identifier PXD010164. Complete selectivity profiles of CZ415 and AZD8055 are available in Supplementary Data 1. Complete matrisome and collagen-modifying protein data is available in Supplementary Data 2. All remaining data will be available from the corresponding author upon reasonable request.
