# Author Correction: The mTORC1/4E-BP1 axis represents a critical signaling node during fibrogenesis

**DOI:** 10.1038/s41467-020-18621-3

**Published:** 2020-09-11

**Authors:** Hannah V. Woodcock, Jessica D. Eley, Delphine Guillotin, Manuela Platé, Carmel B. Nanthakumar, Matteo Martufi, Simon Peace, Gerard Joberty, Daniel Poeckel, Robert B. Good, Adam R. Taylor, Nico Zinn, Matthew Redding, Ellen J. Forty, Robert E. Hynds, Charles Swanton, Morten Karsdal, Toby M. Maher, Andrew Fisher, Giovanna Bergamini, Richard P. Marshall, Andy D. Blanchard, Paul F. Mercer, Rachel C. Chambers

**Affiliations:** 1 Centre for Inflammation and Tissue Repair, UCL Respiratory, Rayne Building, University College London, London, WC1E 6JF UK; 2 Fibrosis Discovery Performance Unit, Respiratory Therapy Area, Medicines Research Centre, GlaxoSmithKline R&D, Gunnels Wood Road, Stevenage, SG1 2NY UK; 3 Target Sciences, Medicines Research Centre, GlaxoSmithKline R&D, Gunnels Wood Road, Stevenage, SG1 2NY UK; 4 Cellzome, a GSK Company, Meyershofstrasse 1, 69117 Heidelberg, Germany; 5 CRUK Lung Cancer Centre of Excellence, UCL Cancer Institute, University College London, London, WC1E 6DD UK; 6 Cancer Evolution and Genome Instability Laboratory, The Francis Crick Institute, London, NW1 1AT UK; 7 Nordic Bioscience, Herlev, 2730 Denmark; 8 Fibrosis Research Group, Inflammation, Repair & Development Section, NHLI, Imperial College, London, SW3 6LY UK; 9 Newcastle Fibrosis Research Group, Newcastle University Translational and Clinical Research Institute, Newcastle upon Tyne, UK

**Keywords:** Transforming growth factor beta, TOR signalling, Respiratory tract diseases

Correction to: *Nature Communications* 10.1038/s41467-018-07858-8, published online 2 January 2019.

The original version of this article omitted from the author list the 19th author Andrew Fisher, who is from the Newcastle Fibrosis Research Group, Newcastle University Translational and Clinical Research Institute, Newcastle upon Tyne, UK. Consequently, the following was added to the Acknowledgements: ‘A.J.F. acknowledges funding support via a collaborative framework agreement between GSK and Newcastle University and from the NIHR Newcastle Biomedical Research Centre (Newcastle BRC).’ Additionally, the following was added to the Author Contributions: ‘A.J.F. obtained ethical approvals, consented patients and provided patient tissue and reviewed and approved the final manuscript.’ Finally, the following was added to the Competing Interests: ‘A.J.F. has no declarations directly related to this manuscript. His institution has received industry-academic research funding from GlaxoSmithKline R&D, Genentech, Pfizer and Nuformix. His institution has received consultancy fees from Mallinckrodt Pharmaceuticals.’ This has been corrected in both the PDF and HTML versions of the article.

