# Supplementary material for: The mTORC1/4E-BP1 axis represents a critical signaling node during fibrogenesis
Source: Nat Commun. 2019 Jan 2;10:6. doi: 10.1038/s41467-018-07858-8 (PMC6315032; doi:10.1038/s41467-018-07858-8)

The mTORC1/4E-BP1 axis represents a critical signalling node during fibrogenesis

Woodcock, Eley et al.

List of Supplementary materials

Supplementary methods

Supplementary Table 1. Antibodies used for Western blotting.

Kinobead assay

Matrisome proteomics methods

- Proteomic sample preparation
- LC-MS/MS analysis
- Protein Identification and quantification
- Statistical analysis of MS data
- MS data representation

Supplementary Tables and Figures

Supplementary Table 2. pIC_{50} in recombinant assays for pharmacological inhibitors used in fibroblast cultures and tissue slice experiments.

Supplementary Figure 1. Structures of pharmacological inhibitors used in fibroblast culture and tissue slice studies.

Supplementary Table 3. pIC_{50} in a macromolecular crowding assay for AZD8055 in human lung fibroblasts derived from control and IPF lungs.

Supplementary Figure 2. MS-based kinase-binding profile of AZD8055 and CZ415 compounds across a set of lipid kinases identified from mixed human cell-line lysates.

Supplementary Table 4. Kinases quantified in profiling of AZD8055 and CZ415.

Supplementary Figure 3. Torin-1 and Compound 1 inhibit TGF-β_1-induced phosphorylation of mTOR substrates in normal human lung fibroblasts.

Supplementary Figure 4. TGF-β_1 induced mTORC1 signalling and collagen response is Akt and ERK independent

Supplementary Figure 5 Effect of doxycycline treatment on untransduced control pHLFs

Supplementary Table 5 Summary statistics of P1NP inhibition in precision-cut lung slices in response to incubation with CZ415

Supplementary Figures 6-15 Uncropped Western blots for main manuscript and supplementary figures

Supplemental Dataset 1 (kinobead profiling)

Supplemental Dataset 2 (expression proteomics)

Supplementary Materials

Supplementary Methods

Antibody	Cat no. (dilution)	Antibody	Cat no. (dilution)
Phospho-SMAD2$^{Ser465/467}$	#3108 (1:1000)	Phospho-4E-BP1^{Ser65}	#9451 (1:2000)
SMAD2	#3103 (1:2000)	Phospho-4E-BP1^{Thr70}	#13396 (1:1000)
Phospho-SMAD3$^{Ser423/425}$	#9520 (1:1000)	4E-BP1	#9644 (1:2000)
SMAD3	#9523 (1:2000)	Phospho-S6$^{Ser235/236}$	#4858 (1:1000)
Phospho-Akt^{Thr308}	#2965 (1:1000)	S6	#2217 (1:1000)
Phospho-Akt^{Ser473}	#4060 (1:1000)	Raptor	#2280 (1:1000)
Akt	#4691 (1:5000)	Rictor	#2114 (1:1000)
Phospho-NDRG1^{Thr346}	#5482 (1:2000)	mTOR	#2983 (1:1000)
NDRG1	#9408 (1:5000)	eIF4E	#9742 (1:1000)
Phospho-PRAS40^{Thr246}	#13175 (1:1000)	eIF4G	#2469 (1:1000)
PRAS40	#2691 (1:1000)	Phospho-TSC2^{Ser939}	#3615 (1:1000)
Phospho-GSK3β^{Ser9}	#5558 (1:1000)	TSC2	#3612 (1:2000)
GSK3β	#12456 (1:1000)	α-tubulin	#9099 (1:4000)
Phospho-p70S6K^{Thr389}	#9234 (1:1000)	Phospho-TSC2 Ser664	#133465 (Abcam) (1:1000)
P70S6K	#9202 (1:1000)	β actin	#A5541 (Sigma) (1:10000)
Phospho-4EBP1$^{Thr37/46}$	#2855 (1:1000)		

Supplementary Table 1 – Antibodies used for Western blotting. All antibodies purchased from Cell Signalling unless otherwise stated.

Kinobead assay

Competition binding assays were performed as described previously by using kinobeads[1,2]. Briefly, 1 mL (5 mg protein) cell extract was pre-incubated with test compound or vehicle for 45 min at 4°C followed by incubation with kinobeads for 1 hour at 4 °C. The beads were washed with lysis buffer and eluted with SDS sample buffer and subjected to SDS gel electrophoresis. Samples were further processed for LC-MS analysis.

Matrisome proteomics methods:

Proteomic sample preparation

PBS-washed cell pellets were frozen in liquid N2 and collectively lysed in 2% SDS, heated for 3 min at 95 °C in a thermomixer (Eppendorf), followed by digestion of DNA with Benzonase at 37 °C for 1.5 h. Lysate was cleared by centrifugation and protein concentration in the supernatant was determined by BCA assay. Proteins were reduced by DTT and alkylated with iodacetamide, separated on 4–12% NuPAGE (Life Technologies) gels and stained with colloidal Coomassie. Gel lanes were cut into three slices covering the entire separation range (~2 cm) and subjected to in-gel digestion. Peptide samples were labelled with 10-plex TMT (TMT10, ThermoFisher Scientific) reagents. The labelling reaction was performed in 40mM triethylammoniumbicarbonate, pH 8.53, at 22 °C and quenched with glycine. Labelled peptide extracts were combined to a single sample per experiment, and subjected to additional fractionation on an Ultimate3000 (Dionex) by using reverse-phase chromatography at pH 12 [1 mm Xbridge column (Waters)], as previously described[3].

LC-MS/MS analysis

Samples were dried in vacuum and resuspended in 0.05 % trifluoroacetic acid in water. Of the sample, 50% was injected into an Ultimate3000 nanoRLSC HPLC (Dionex) coupled to a Q-Exactive Mass Spectrometer (ThermoFisher Scientific). Peptides were trapped on a 5 mm x 300 µm C18 column (Pepmap100, 5 µm, 300 Å, Thermo Fisher Scientific) in water with 0.05 % TFA at 60 °C. Separation was performed on custom 50 cm × 100 µm (ID) reversed-phase columns (Reprosil) at 55°C. Gradient elution was performed from 2% acetonitrile to 40% acetonitrile in 0.1% formic acid and 3.5 % DMSO over 2 hours. Samples were online injected into Q-Exactive plus mass spectrometers operating with a data-dependent top 10 method. MS spectra were acquired by using 70.000 resolution and an ion target of 3x10^6. Higher energy collisional dissociation (HCD) scans were performed with 33% NCE at 35.000 resolution (at m/z 200), and the ion target settings was set to 2x10^5 so as to avoid coalescence[4].

The mass spectrometry proteomics data have been deposited to the ProteomeXchange Consortium via the PRIDE partner repository with the dataset identifier PXD010164[5].

Protein identification and quantification:

Mascot 2.4 (Matrix Science) was used for protein identification by using a 10 p.p.m. mass tolerance for peptide precursors and 20 mD (HCD) mass tolerance for fragment ions. Carbamidomethylation of cysteine residues and TMT modification of lysine residues were set as fixed modifications and methionine oxidation, and N-terminal acetylation of proteins and TMT modification of peptide N-termini were set as variable

modifications. The search database consisted of a customized version of the International Protein Index protein sequence database combined with a decoy version of this database created by using a script supplied by Matrix Science. Unless stated otherwise, we accepted protein identifications as described[4]. Reporter ion intensities were read from raw data and multiplied with ion accumulation times (the unit is milliseconds) so as to yield a measure proportional to the number of ions; this measure is referred to as ion area[4]. Spectra matching to peptides were filtered according to the following criteria: mascot ion score >15, signal-to-background of the precursor ion > 4, and signal-to-interference > 0.5[6]. Fold changes were corrected for isotope purity as described and adjusted for interference caused by co-eluting nearly isobaric peaks as estimated by the signal-to-interference measure[7]. Protein quantification was derived from individual spectra matching to distinct peptides by using a sum-based bootstrap algorithm; 95% confidence intervals were calculated for all protein fold changes that were quantified with more than three spectra[6]. Protein fold changes were only reported for proteins with at least 2 quantified unique peptide (QUP) matches.

Statistical analysis of MS data

Proteins quantified with at least 2 unique peptide matches were divided into bins. The bins are constructed according to the number of quantified spectrum sequence matches. Each bin consists of at least 300 proteins. This data quality–dependent binning strategy is analogous to the procedure described previously[8]. For each protein fold change (FC) a p-value is calculated using a Z-test with a robust estimation of the mean and standard deviation (using the 15.87, 50, and 84.13 percentiles). The

standard deviation is calculated, per bin, from a distribution of proteins log2 transformed fold changes. Subsequently, an adjustment for multiple hypothesis testing was performed on the full data set by using Benjamini-Hochberg (BH) correction[9]. Proteins are considered significantly regulated when p-value <0.05 and ≥30 % change in relative abundance with same direction in both replicates.

MS data representation

Treatment effects were summarised as ratio to DMSO or TGF-β_1 for each condition. Briefly, the log2 fold change for each replicate pair was calculated and the mean taken to represent overall effect. Core matrisome proteins (http://matrisomeproject.mit.edu/other-resources/human-matrisome/) and proteins involved in collagen synthesis and degradation (https://reactome.org/PathwayBrowser/#/R-HSA-1650814) were filtered for a |FC| >1.2 (treatment vs vehicle alone) for heatmap representation. The heatmap was generated in Rv3.3.3 using gplots (https://cran.r-project.org/web/packages/gplots/index.html) and protein dendrogram was sorted using the R package dendsort (https://cran.r-project.org/web/packages/dendsort/index.html).

Supplementary Figures

Compound Name	PI3K				mTORC1/2*		DNA-PK	Target	HSA%
	α	β	γ	δ	Biochemical (FRAP1)	Kinobeads			HPLC
**CZ415**[10]	5.5	5.1	5.1	5.2	8.3	8.0	5.8		91.4
**Torin 1**[11]	6.8	5.5	7.0	6.2	ND	7.2	7.6		96.5
**AZD8055**[12]	5.5	4.7	5.3	5.8	8.2	8.5	5.8		92.0
**1 (Wyeth)**[13]	5.2	5.2	5.2	5.3	7.3	7.0	7.3		96.6
**2 (Genentech)**[14]	7.4	6.9	7.3	7.2	<4.3	<4.0	6.2		95.5
**MK2206**[15]	4.4	4.7	4.4	4.8	<4.3	ND	<4.1	AKT: 8.3**	93.0
**GSK2334470**[16]	4.9	4.6	4.9	4.9	<10% inhibition at 1 µM	<5.3	-	PDK1: 8.6†	94.3

Supplementary Table 2. pIC_{50} in recombinant assays for pharmacological inhibitors (GlaxoSmithKline recombinant data unless otherwise stated). *mTORC1/2 potency data were obtained from i) FRAP1 recombinant biochemical assay (similar to [10]) and ii) Kinobead chemoproteomic binding assay with antibody readout (similar to [11]). ** Data from [12] † Data from [13]. ND, not determined. HSA%: human serum albumin binding (%).

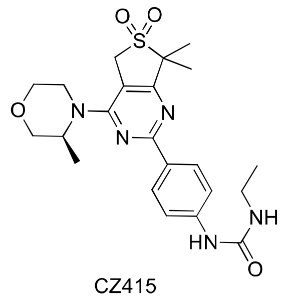

CZ415

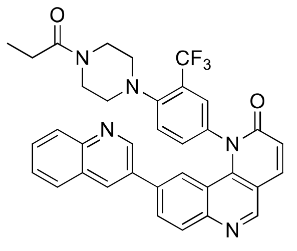

Torin 1

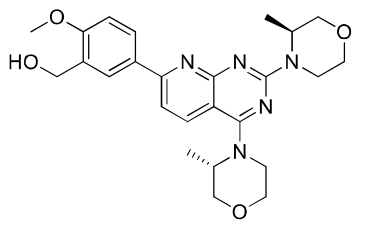

AZD8055

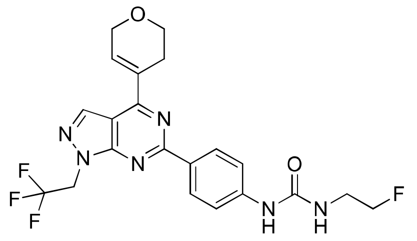

(1)

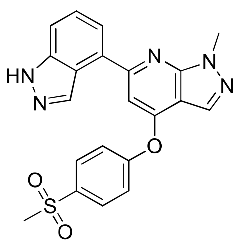

(2)

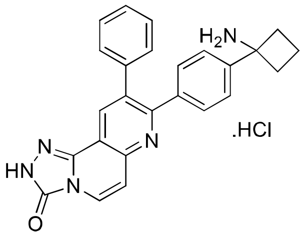

MK2206

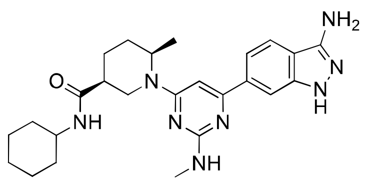

GSK2334470

Supplementary Figure 1. Structures of pharmacological inhibitors used in fibroblast culture and tissue slice studies.

	Donor ID	Log [M] = pIC_{50}
**Control lines**	Donor 1	-7.43
	Donor 2	-6.82
	Donor 3	-6.43
	Donor 4	-6.98
	Donor 5	-6.43
**IPF lines**	Donor 1	-6.70
	Donor 2	-7.01
	Donor 3	-6.70
	Donor 4	-6.22
	Donor 5	-6.93

Supplementary Table 3: pIC_{50} in a macromolecular crowding assay for AZD8055 in human lung fibroblasts derived from control and IPF donors.

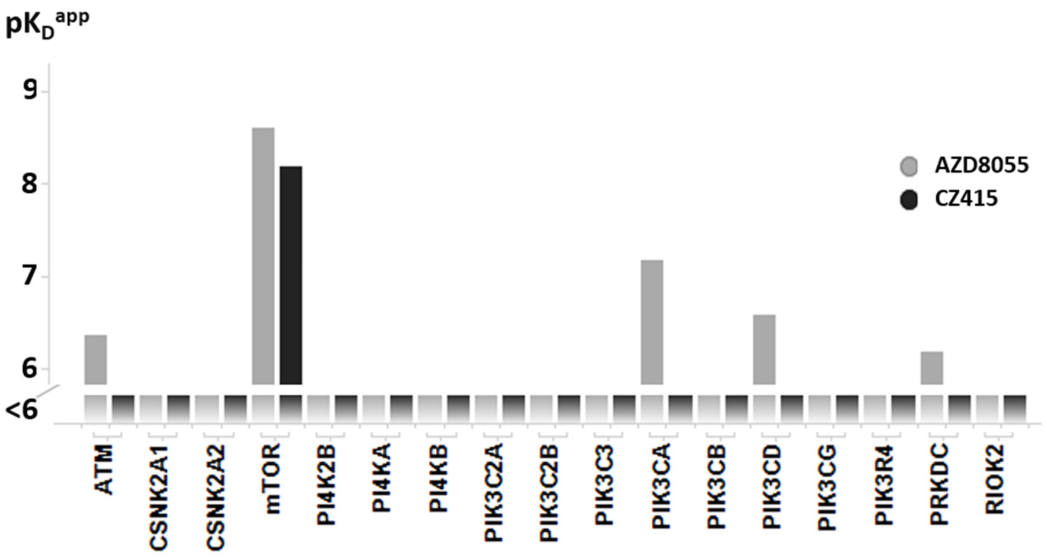

Supplementary Figure 2. MS-based kinase-binding profile of AZD8055 and CZ415 compounds across a set of lipid kinases identified from mixed human cell-line lysates. The bars indicate pKd app values defined as the concentration of drug at which half-maximal competition of binding is observed, corrected by the influence of the immobilized ligand on the binding equilibrium using the Cheng−Prusoff equation[17]. Grey bars = AZD8055, black bars = CZ415.

AAK1	BUB1	CRKRS	EPHB3	IKBKE	MAP2K5	MAPKAPK5	NTRK1	PLK1	RPS6KA6	TBK1
ABL1	CABC1	CSF1R	EPHB4	ILK	MAP2K6	MARK1	OXSR1	PLK4	RPS6KB1	TEC
ABL2	CAMK2B	CSK	EPHB6	INSR	MAP3K1	MARK2	PAK4	PRKAA1	SGK3	TEK
ACVR1	CAMK2D	CSNK1A1	ERBB4	IRAK1	MAP3K11	MARK3	PCTK1	PRKAA2	SIK2	TESK1
ACVR1B	CAMK2G	CSNK1D	ERN1	IRAK3	MAP3K15	MARK4	PCTK2	PRKCA	SLK	TESK2
ACVR2A	CAMKK1	CSNK1E	FER	IRAK4	MAP3K2	MAST2	PCTK3	PRKCB	SNRK	TGFBR1
ACVR2B	CAMKK2	CSNK2A1	FES	ITK	MAP3K3	MAST3	PDGFRB	PRKCD	SRC	TGFBR2
ADCK1	CDC2	CSNK2A2	FGFR1	JAK1	MAP3K4	MASTL	PDIK1L	PRKCI	STK10	TNIK
AKT1	CDC2L5	DAPK1	FGFR2	JAK2	MAP3K5	MELK	PDPK1	PRKCQ	STK11	TNK1
AKT2	CDC42BPA	DAPK3	FGFR3	KDR	MAP3K6	MERTK	PFTK1	PRKCZ	STK16	TNK2
ARAF	CDC42BPB	DDR1	FGR	QSK	MAP3K7	MET	PHKG2	PRKD1	STK17A	TP53RK
AURKA	CDC7	DSTYK	FLT4	MLK4	MAP4K2	MINK1	PIK3C3	PRKD2	STK17B	TTK
AURKB	CDK10	DYRK1A	FRK	KIT	MAP4K3	MKNK1	PIK3R4	PRKD3	STK3	TYK2
AXL	CDK2	EGFR	FYN	KSR1	MAP4K4	MKNK2	PIM1	PRKG1	STK33	TYRO3
BCR	CDK5	EIF2AK1	GAK	LATS1	MAP4K5	MST1R	PIM2	PRKX	STK35	ULK1
BMP2K	CDK7	EIF2AK4	GSK3A	LIMK1	MAPK1	MYLK	PIM3	PTK2	STK38	ULK3
BMPR1A	CDK8	EPHA1	GSK3B	LIMK2	MAPK11	MYLK3	PIP4K2A	PTK2B	STK38L	WEE1
BMPR1B	CDK9	EPHA2	HCK	MAST4	MAPK14	NEK1	PIP4K2B	RIOK2	STK39	YES1
BMPR2	CDKL5	EPHA3	HIPK1	LRRK2	MAPK3	NEK2	PIP4K2C	RIPK2	STK4	ZAK
BMX	CHEK1	EPHA4	HIPK2	LYN	MAPK6	NEK3	PIP5K3	RPS6KA1	STRADA	
BRAF	CHEK2	EPHA5	HIPK3	MAP2K1	MAPK7	NEK6	PKMYT1	RPS6KA2	SYK	
BRSK1	CHUK	EPHA7	ICK	MAP2K2	MAPK8	NEK7	PKN1	RPS6KA3	TAOK1	
BRSK2	CIT	EPHB1	IGF1R	MAP2K3	MAPK9	NEK9	PKN2	RPS6KA4	TAOK2	
BTK	CLK1	EPHB2	IKBKB	MAP2K4	MAPKAPK3	NLK	PKN3	RPS6KA5	TAOK3	

Supplementary Table 4. List of all protein kinases quantified in MS-based kinase-binding profiles of AZD8055 and CZ415 compounds identified from mixed human cell-line lysates. None of the kinases were inhibited by either compound with a pIC_{50} >5.

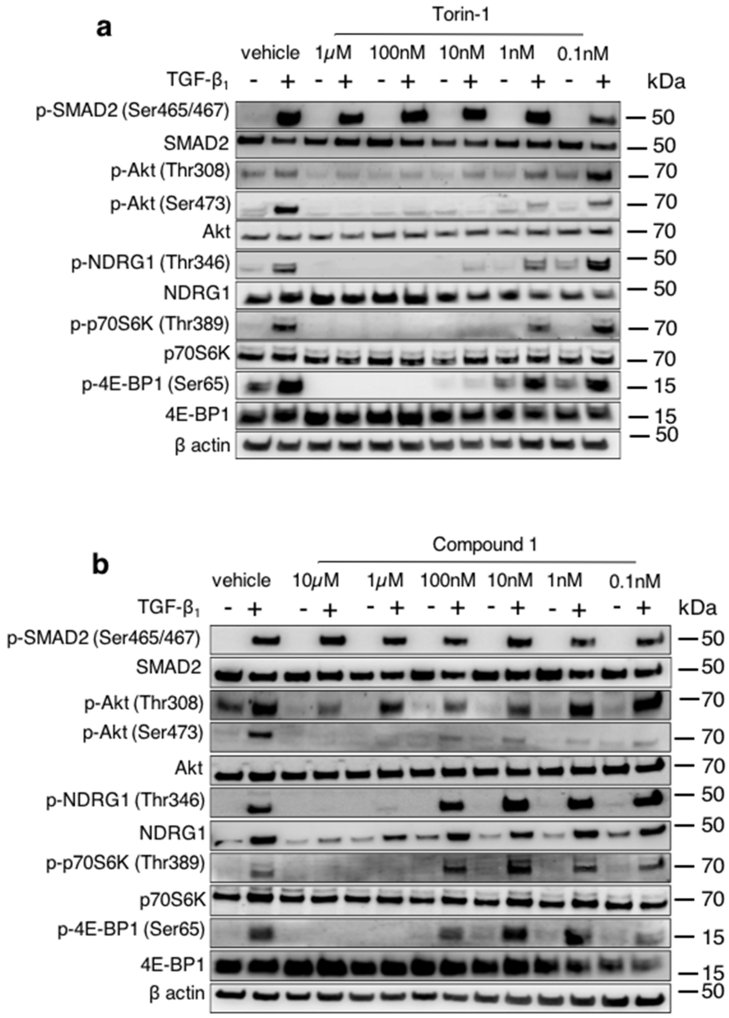

Supplementary Figure 3. Torin-1 and Compound 1 inhibit TGF-β$_1$ -induced phosphorylation of mTOR substrates in control pHLFs. pHLFs were pre-incubated with vehicle (0.1% DMSO) or increasing concentrations of Torin-1 (a) or Compound 1 (b) prior to stimulation with TGF-β$_1$ (1ng/ml). Cells were lysed at 1 hour to assess Smad phosphorylation or at 12 hours to assess phosphorylation of specified proteins. Data are representative of 3 replicate wells per condition.

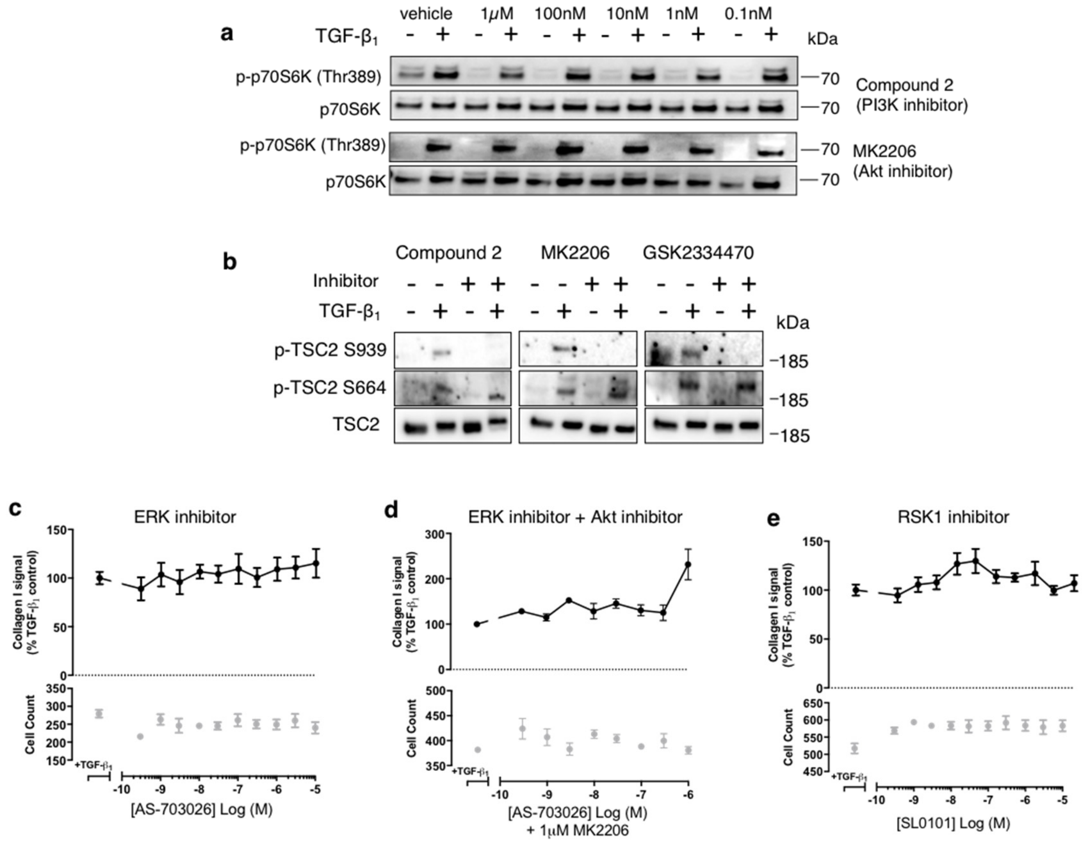

Supplementary Figure 4. TGF-β$_1$ induced mTORC1 signalling and collagen response is Akt- and ERK- independent. Control pHLFs were pre-incubated with vehicle (0.1% DMSO) or increasing concentrations of Compound 2 (PI3K inhibitor) or MK2206 (Akt inhibitor) (a) or 1μM Compound 2, MK2206 or GSK2334470 (PDK1 inhibitor) (b) prior to stimulation with TGF-β$_1$ (1ng/ml). Cells were lysed 12 hours later and the phosphorylation of specified phosphoproteins assessed by Western blot. Additionally, control pHLFs were pre-incubated with vehicle (0.1% DMSO) or increasing concentrations of AS-703026 (ERK inhibitor) alone (c) or in combination with 1μM MK2206 (Akt inhibitor) (d), or increasing concentrations of SL0101 (RSK1 inhibitor) (e) and stimulated with TGF-β$_1$ (1ng/ml) for 72 hours with collagen deposition assessed in the macromolecular crowding assay. Data are expressed as collagen I signal calculated as a percentage of the TGF-β$_1$ -treated control (*n*=4 fields of view imaged per well) and cell counts obtained by staining nuclei with DAPI. Each data point shown is mean +/- SEM of 4 replicate wells per condition and is representative of 3 independent experiments.

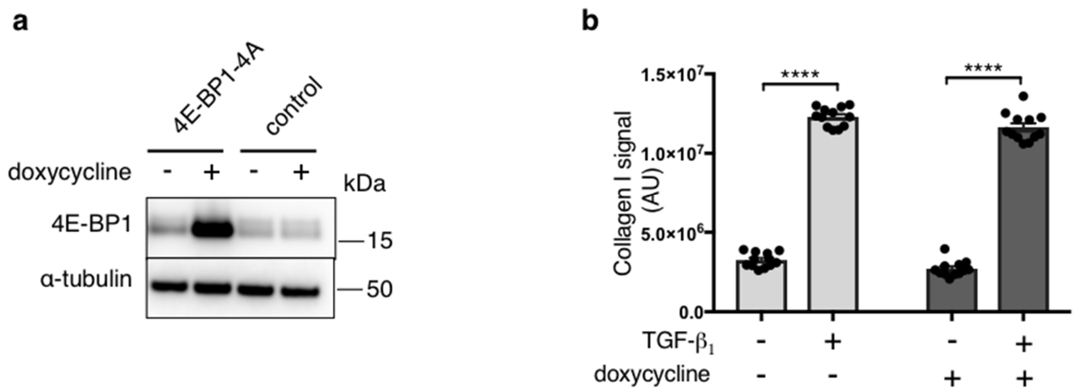

Supplementary Figure 5. Effect of doxycycline treatment on untransduced control pHLFs. pHLFs expressing 4E-BP1-4A dominant negative phosphomutant and untransduced pHLFs were treated with doxycycline 1µg/ml for 24 hours prior to cell lysis and analysis of 4E-BP1 expression using Western blot (a). Additionally, untransduced pHLFs were treated with doxycycline for 24 hours prior to TGF-β_1 stimulation for 72 hours with collagen deposition assessed by macromolecular crowding assay. Data are expressed as collagen I signal (n=4 fields of view imaged per well). Data are presented as mean +/- SEM (*n*=12). Differences between groups were evaluated with two-way ANOVA with Tukey multiple comparison testing. ****=p<0.0001.

	[CZ415]	
	10µM	1µM
Donor 1	97.4	97.6
Donor 2	91.1	86.7
Donor 3	84.1	76.1
Donor 4	67.4	63.2
Donor 5	92.1	88.8
Donor 6	81.9	71.6

Average	85.6	80.7
SD	10.6	12.6
95% CI	8.5	10.1
n	6	6

Supplementary Table 5. Summary statistics of P1NP inhibition in precision-cut lung slices in response to incubation with CZ415. Data presented are average percent inhibition of P1NP levels (ng/ml) relative to the vehicle control within each donor. Summary statistics (mean, standard deviation, 95% confidence interval and number of donors) are provided.

Supplementary References

1. Bantscheff, M. *et al.* Quantitative chemical proteomics reveals mechanisms of action of clinical ABL kinase inhibitors. *Nat. Biotechnol.* **25,** 1035–1044 (2007).
2. Werner, T. *et al.* High-Resolution Enabled TMT 8-plexing. *Anal. Chem.* **84,** 7188–7194 (2012).
3. Savitski, M. M. *et al.* Multiplexed Proteome Dynamics Profiling Reveals Mechanisms Controlling Protein Homeostasis. *Cell* **173,** 260–274.e25 (2018).
4. Werner, T. *et al.* Ion Coalescence of Neutron Encoded TMT 10-Plex Reporter Ions. *Anal. Chem.* **86,** 3594–3601 (2014).
5. Vizcaíno, J. A. *et al.* 2016 update of the PRIDE database and its related tools. *Nucleic Acids Res.* **44,** D447–D456 (2016).
6. Savitski, M. M. *et al.* Measuring and Managing Ratio Compression for Accurate iTRAQ/TMT Quantification. *J. Proteome Res.* **12,** 3586–3598 (2013).
7. Savitski, M. M. *et al.* Delayed Fragmentation and Optimized Isolation Width Settings for Improvement of Protein Identification and Accuracy of Isobaric Mass Tag Quantification on Orbitrap-Type Mass Spectrometers. *Anal. Chem.* **83,** 8959–8967 (2011).
8. Cox, J. & Mann, M. MaxQuant enables high peptide identification rates, individualized p.p.b.-range mass accuracies and proteome-wide protein quantification. *Nat. Biotechnol.* **26,** 1367–1372 (2008).
9. Benjamini, Y. & Hochberg, Y. Controlling the False Discovery Rate: A Practical and Powerful Approach to Multiple Testing. *Journal of the Royal Statistical Society. Series B (Methodological)* **57,** 289–300 (1995).
10. Cansfield, A. D. *et al.* CZ415, a Highly Selective mTOR Inhibitor Showing in Vivo Efficacy in a Collagen Induced Arthritis Model. *ACS Med. Chem. Lett.* **7,** 768–73 (2016).
11. Thoreen, C. C. *et al.* An ATP-competitive mammalian target of rapamycin inhibitor reveals rapamycin-resistant functions of mTORC1. *J. Biol. Chem.* **284,** 8023–32 (2009).
12. Pike, K. G. *et al.* Optimization of potent and selective dual mTORC1 and mTORC2 inhibitors: the discovery of AZD8055 and AZD2014. *Bioorg. Med. Chem. Lett.* **23,** 1212–6 (2013).
13. Kaplan, J. *et al.* Discovery of 3,6-dihydro-2H-pyran as a morpholine replacement in 6-aryl-1H-pyrazolo[3,4-d]pyrimidines and 2-arylthieno[3,2-d]pyrimidines: ATP-competitive inhibitors of the mammalian target of rapamycin (mTOR). *Bioorg. Med. Chem. Lett.* **20,** 640–3 (2010).
14. Staben, S. T. *et al.* Structure-based optimization of pyrazolo-pyrimidine and -pyridine inhibitors of PI3-kinase. *Bioorg. Med. Chem. Lett.* **20,** 6048–51 (2010).
15. Yap, T. A. *et al.* First-in-Man Clinical Trial of the Oral Pan-AKT Inhibitor MK-2206 in Patients With Advanced Solid Tumors. *J. Clin. Oncol.* **29,** 4688–4695 (2011).
16. Medina, J. R. *et al.* Structure-based design of potent and selective 3-phosphoinositide-dependent kinase-1 (PDK1) inhibitors. *J. Med. Chem.* **54,** 1871–95 (2011).
17. Yung-Chi, C. & Prusoff, W. H. Relationship between the inhibition constant (KI) and the concentration of inhibitor which causes 50 per cent inhibition (I50) of an enzymatic reaction. *Biochem. Pharmacol.* **22,** 3099–3108 (1973).

Supplementary Figure 6: Figure 1a uncropped western blots (TGF-β1 time course)

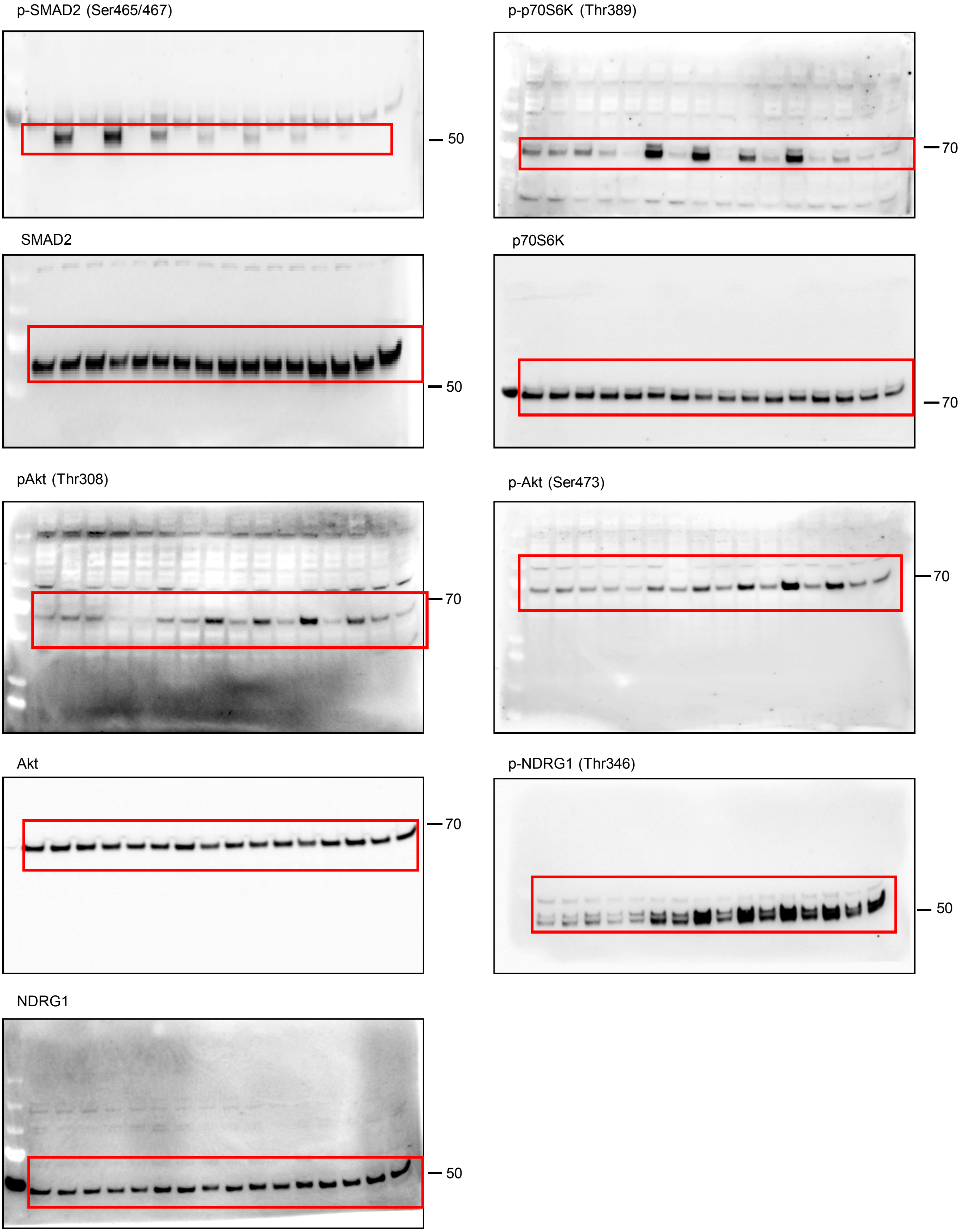

Supplementary Figure 6: Figure 1a uncropped western blots (continued)

p-4E-BP1 (Ser65)

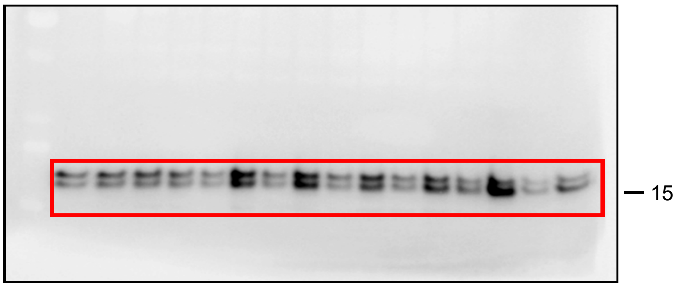

β actin

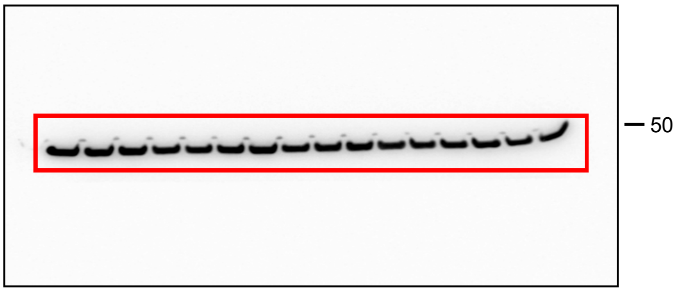

4E-BP1

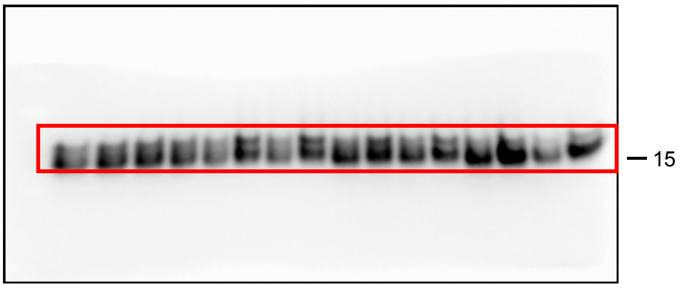

Supplementary Figure 7: Figure 2b uncropped western blots (Compound 2 (PI3K inhibitor))

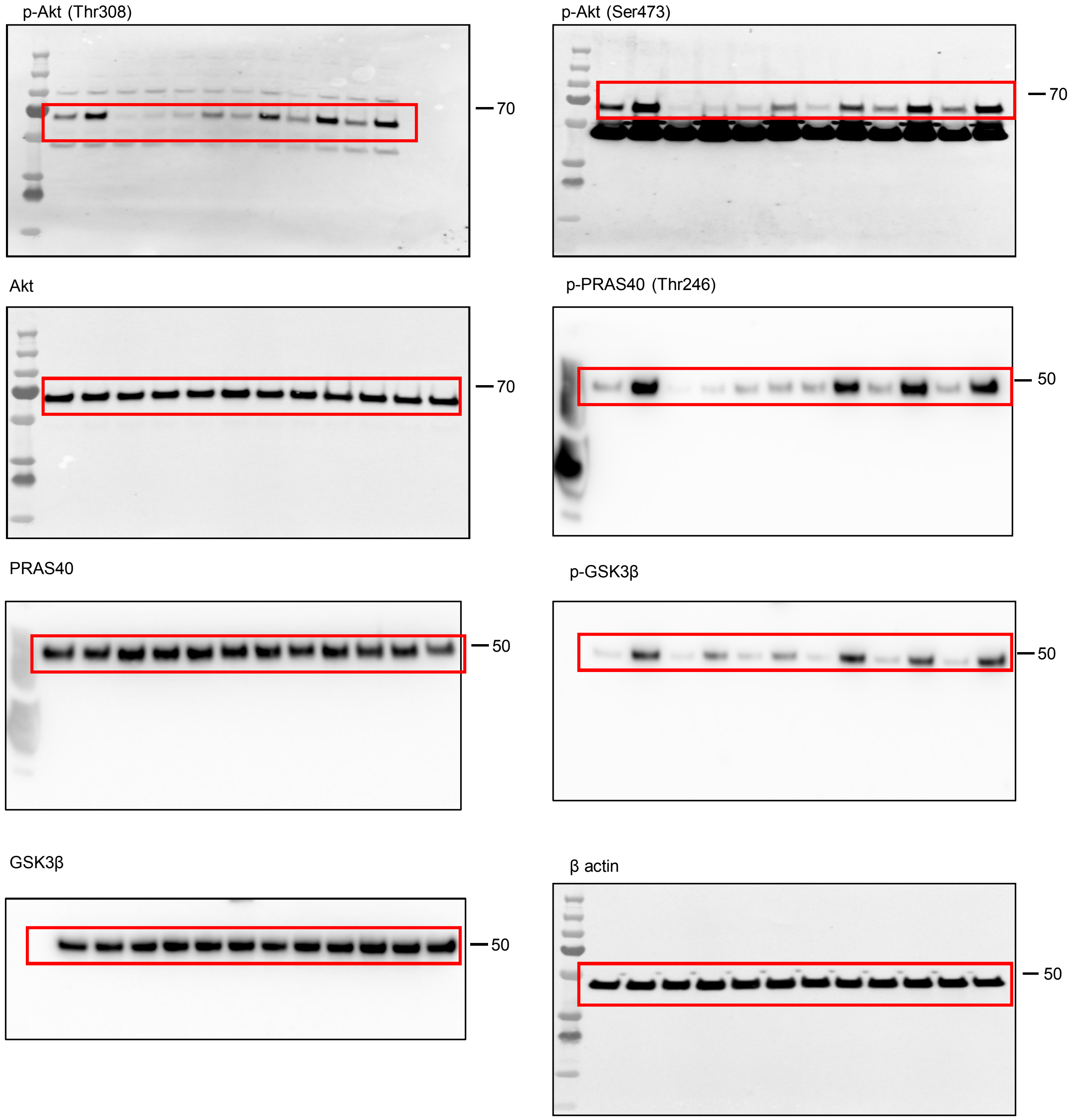

Supplementary Figure 7: Figure 2d uncropped western blots (MK2206 (Akt inhibitor))

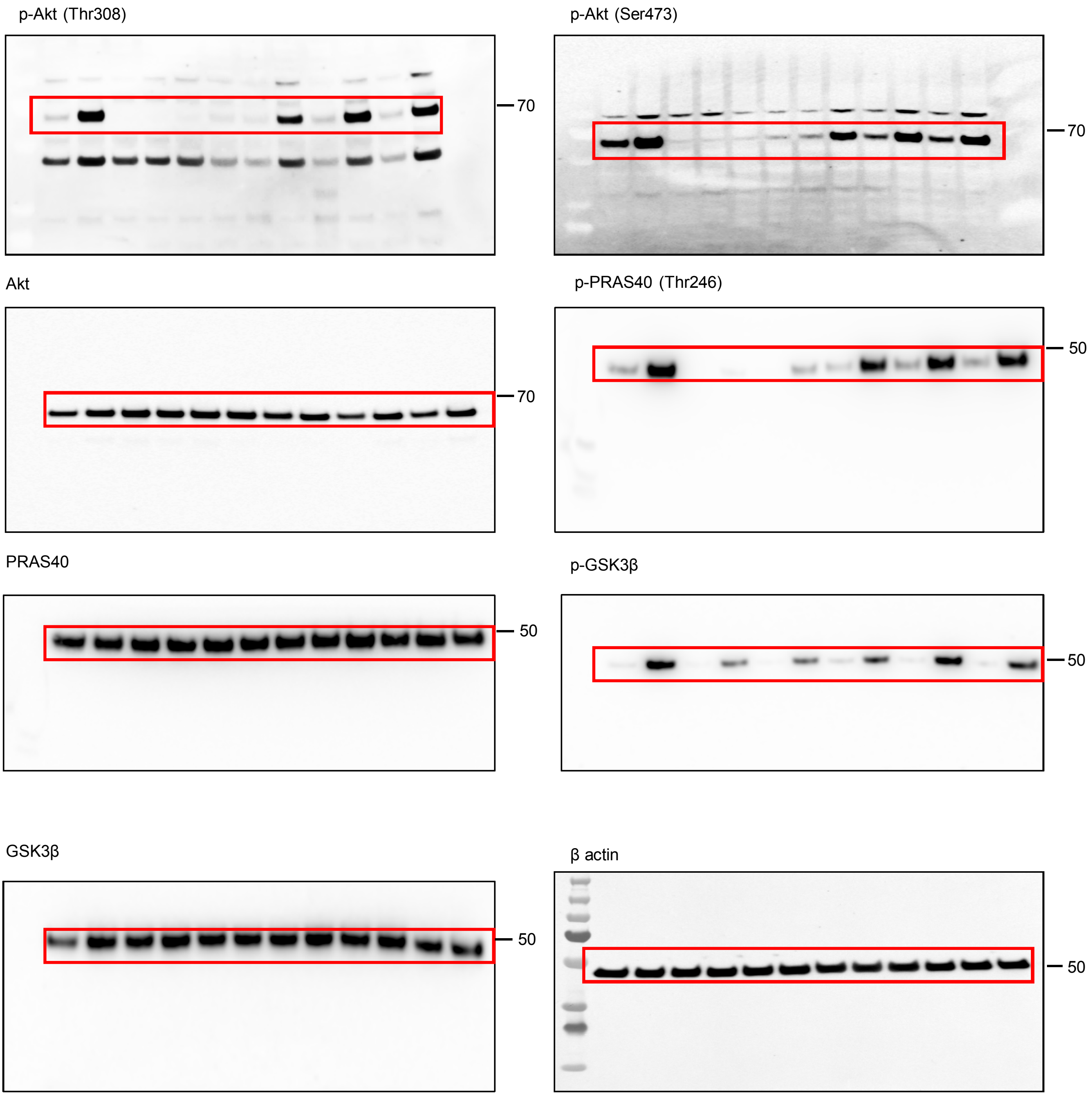

Supplementary Figure 7: Figure 2f uncropped western blots (GSK2334470 (PDK1 inhibitor))

p-Akt (Thr308)

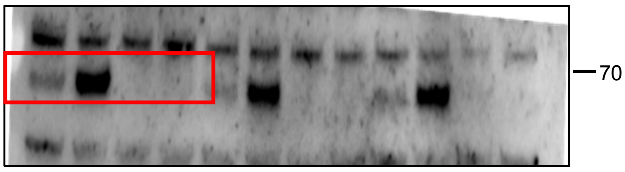

p-Akt (Ser473)

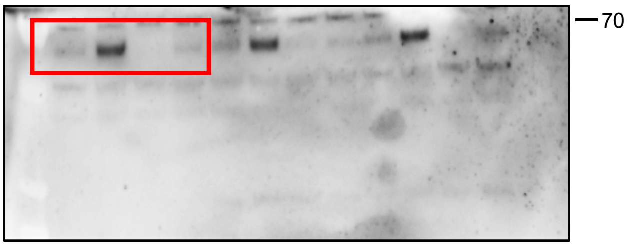

Akt

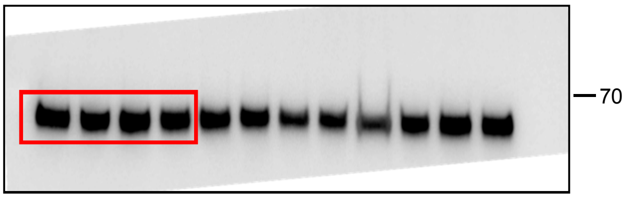

p-NDRG1 (Thr346)

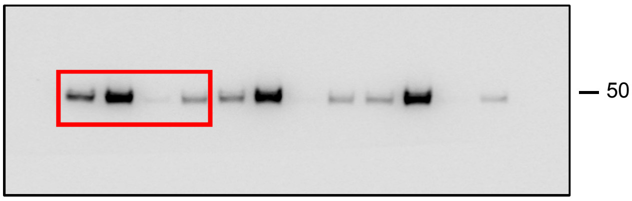

NDRG1

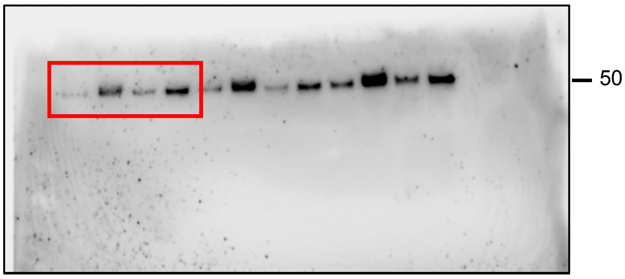

p-PRAS40 (Thr246)

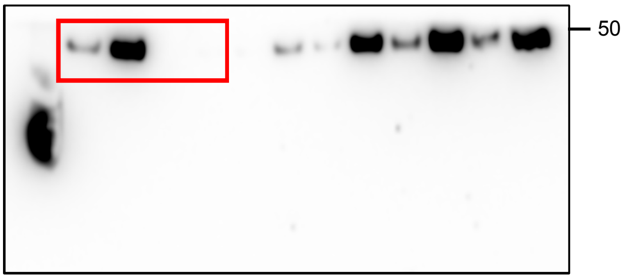

PRAS40

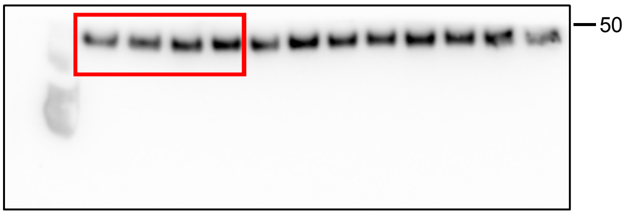

p-GSK3β

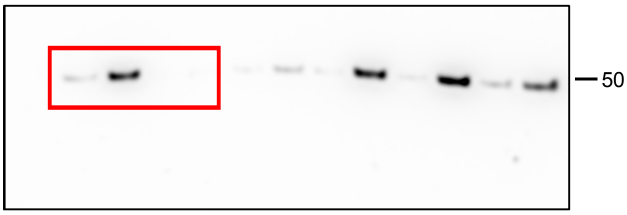

GSK3β

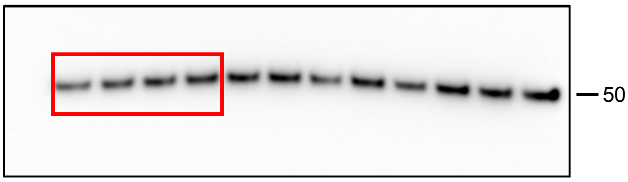

β actin

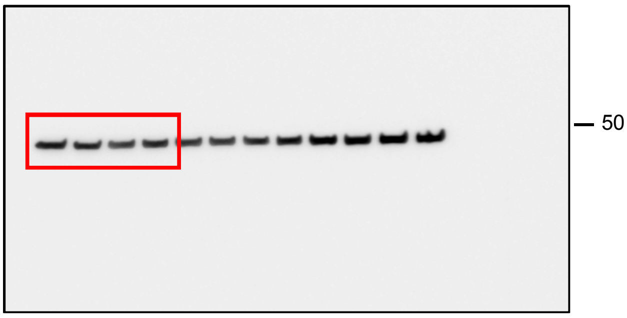

Supplementary Figure 8: Figure 3a uncropped western blots (AZD8055)

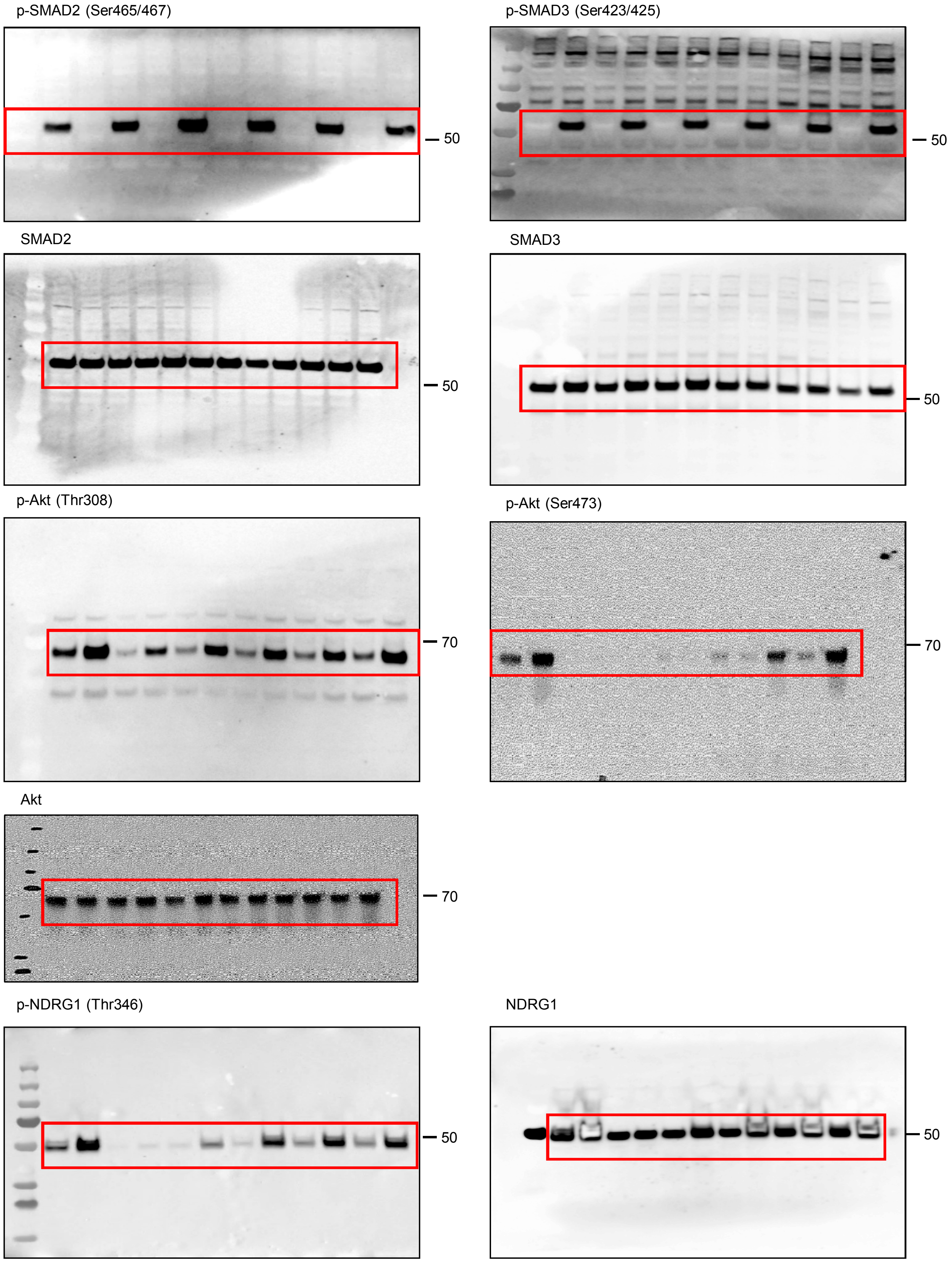

Supplementary Figure 8: Figure 3a uncropped western blots (continued)

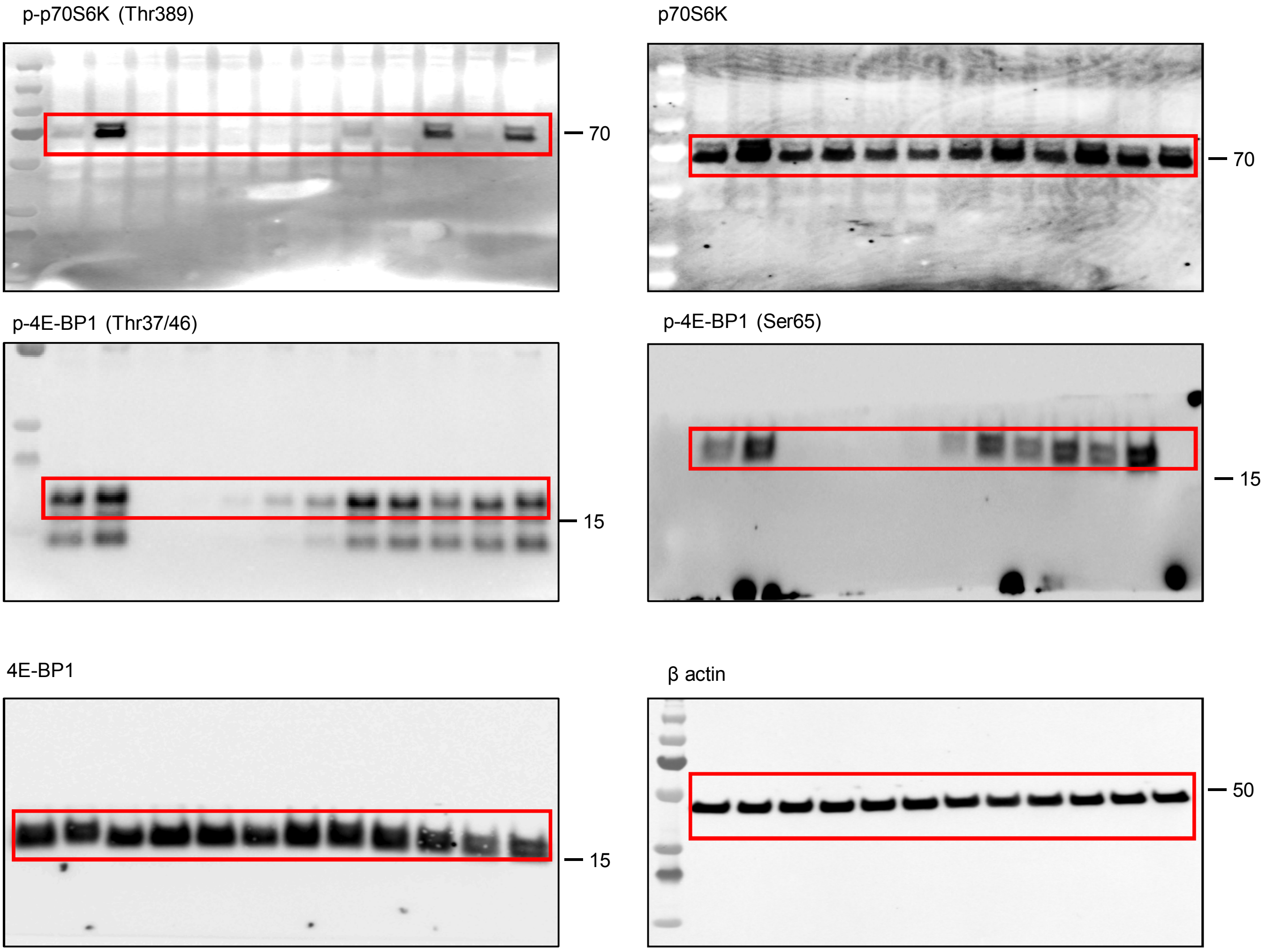

Supplementary Figure 9: Figure 4a uncropped western blots

SMAD3

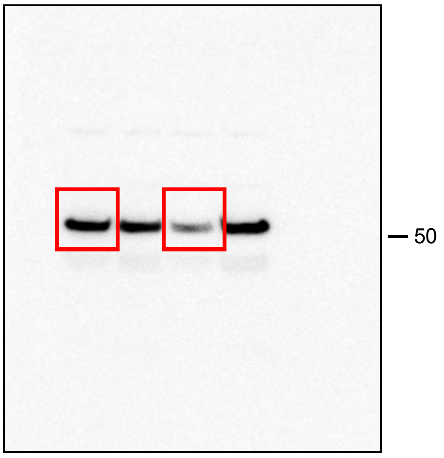

β actin

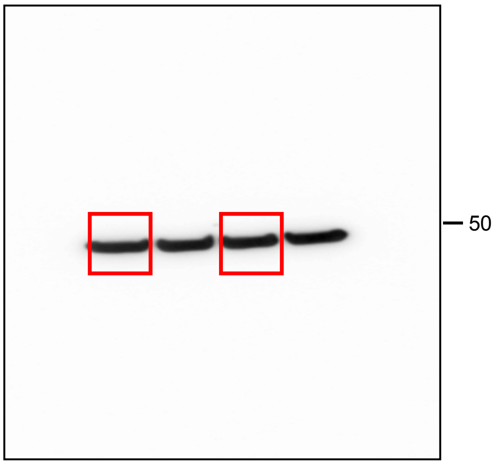

Supplementary Figure 9: Figure 4b uncropped western blots

p-p70S6K (Thr389)

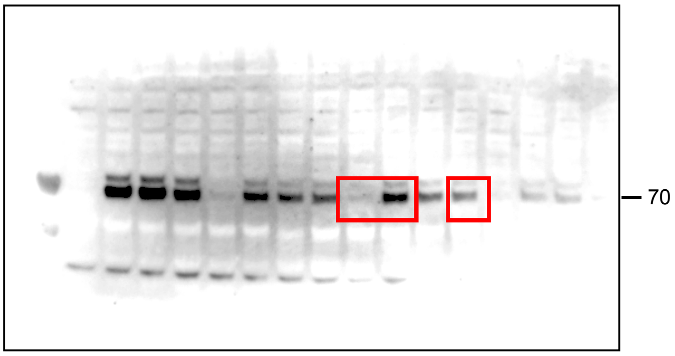

p70S6K

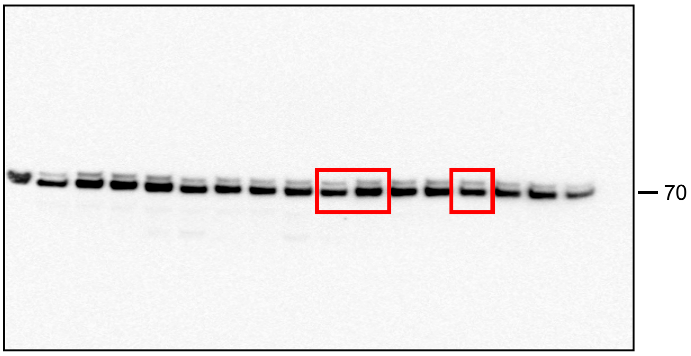

p-4E-BP1 (Ser65)

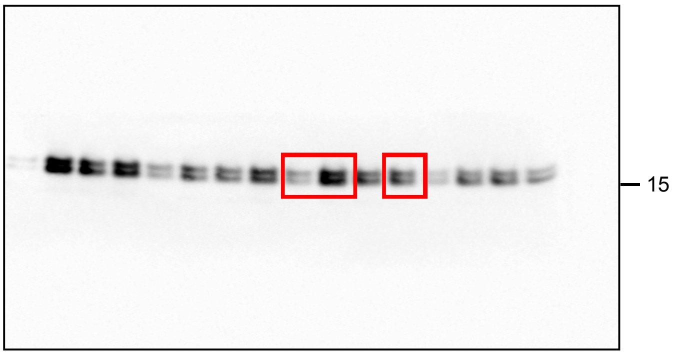

4E-BP1

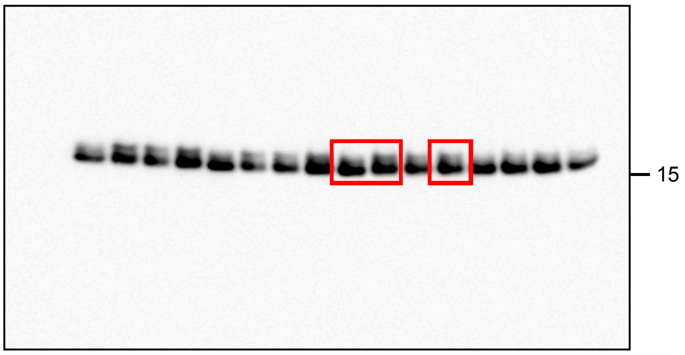

Supplementary Figure 10: Figure 5c uncropped western blots (rapamycin)

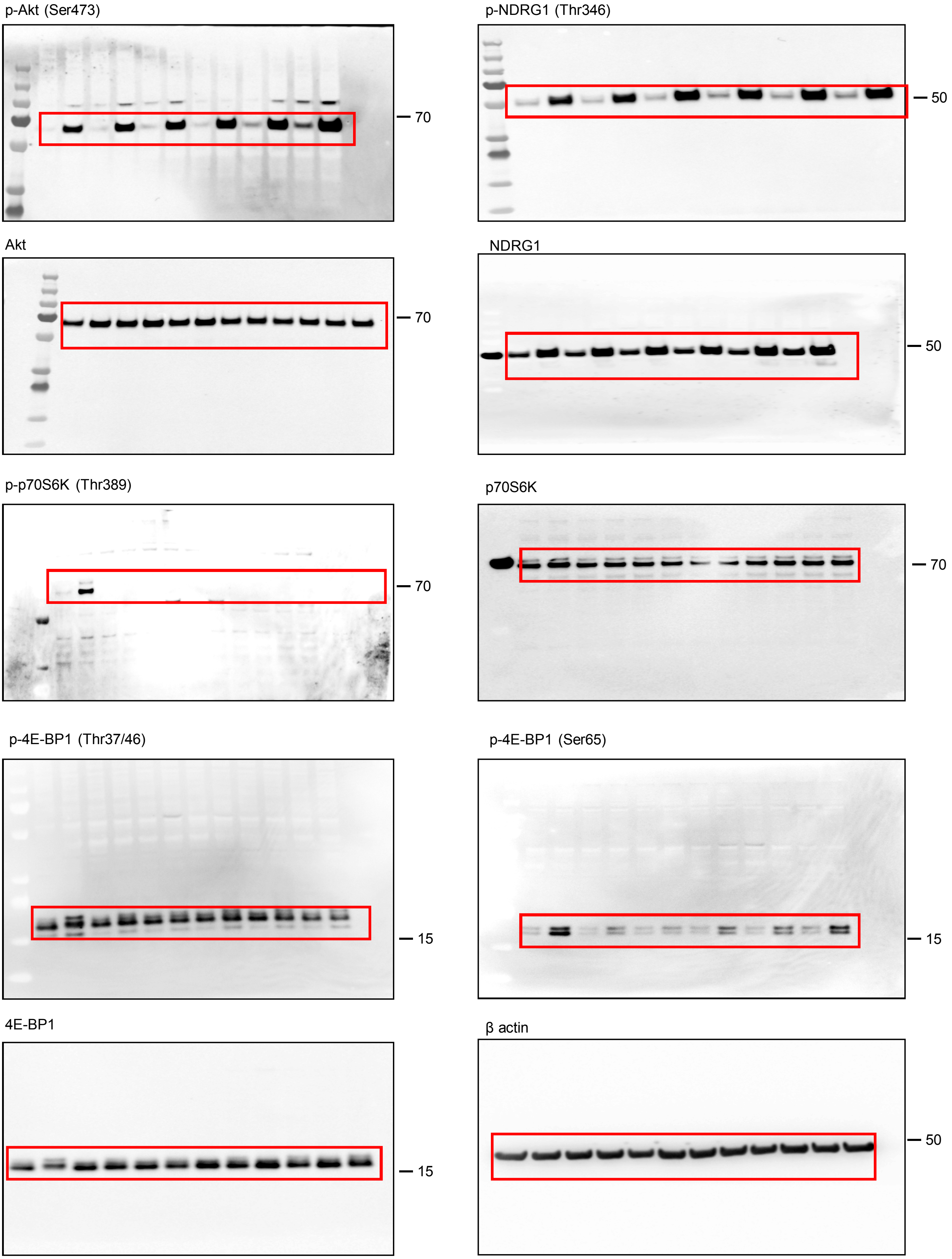

Supplementary Figure 10: Figure 5d uncropped western blots (LY2584702)

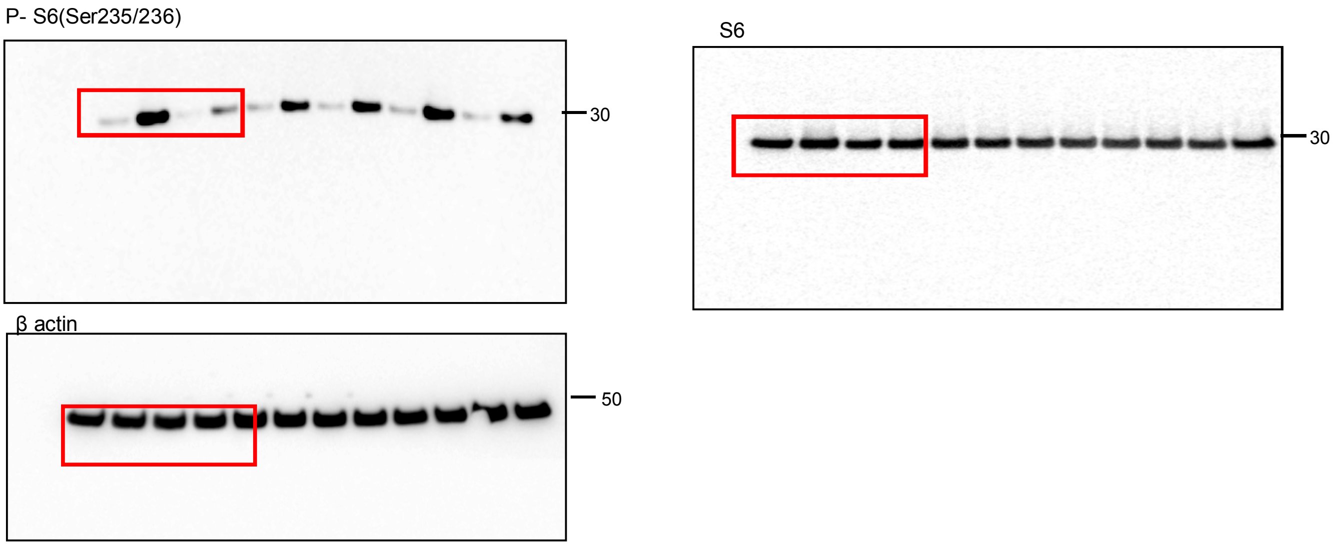

Supplementary Figure 10: Figure 5f uncropped western blots (GSK2334470)

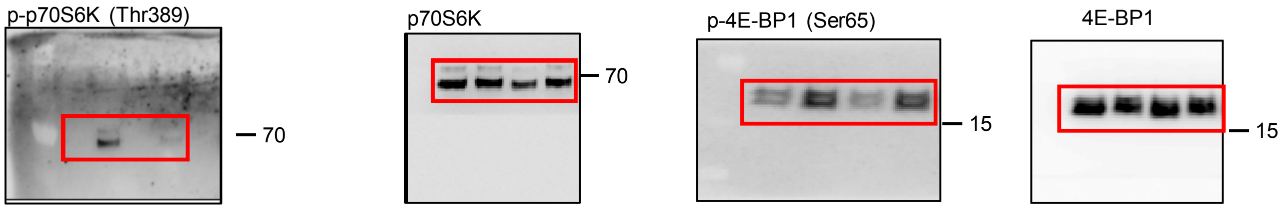

Supplementary Figure 10: Figure 5g uncropped western blots (cap pull down)

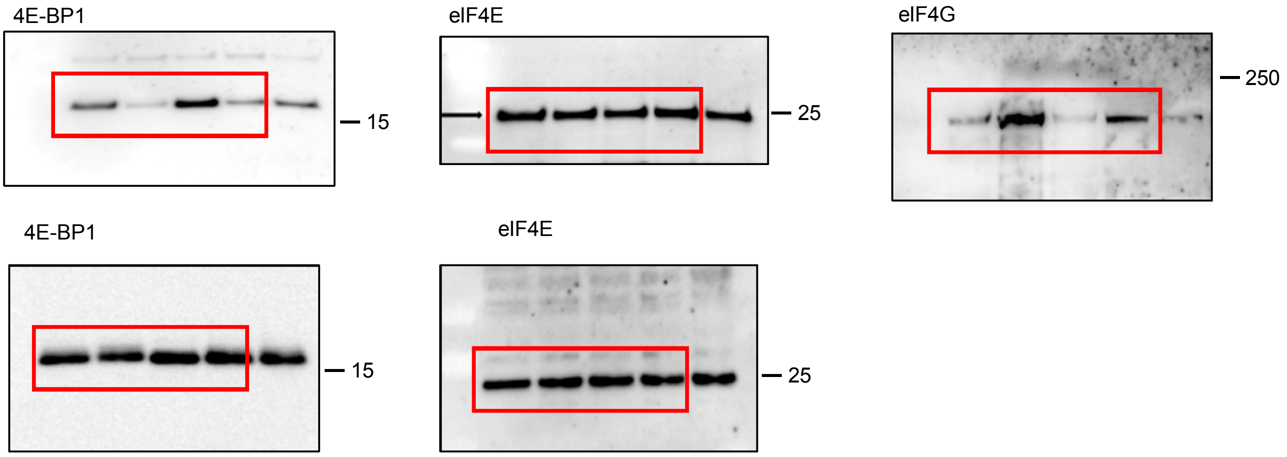

Supplementary Figure 11: Figure 6a uncropped western blots (4E-BP1 siRNA pHLFs)

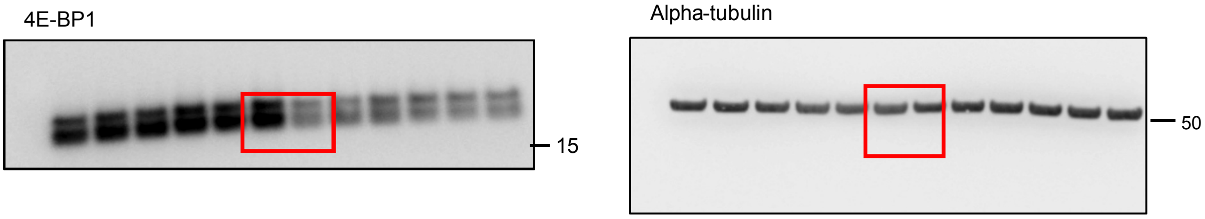

Supplementary Figure 11: Figure 6c uncropped western blots (cap pull down phosphomutant) - immunoprecipitation

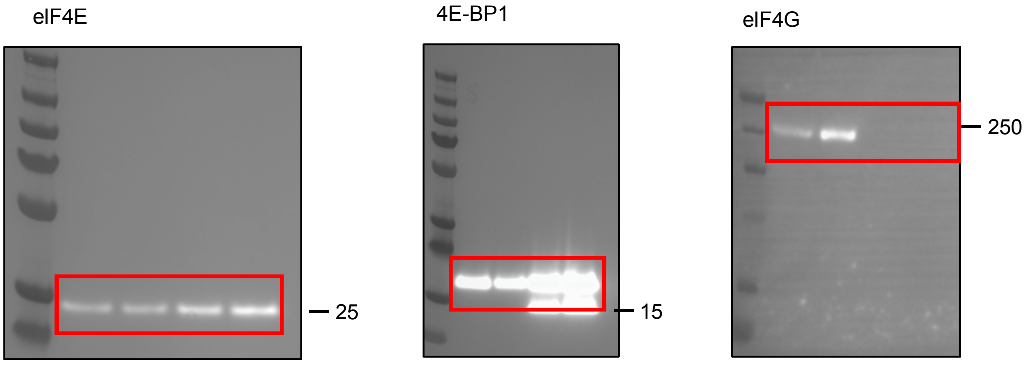

Supplementary Figure 11: Figure 6c uncropped western blots (cap pull down phosphomutant) - Lysate

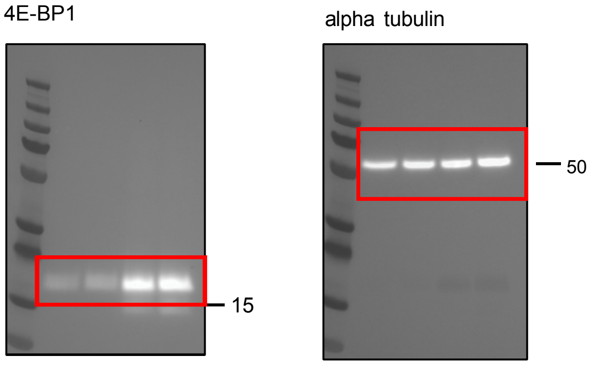

Supplementary Figure 12: Figure 9a uncropped western blots (CAF CRISPR)

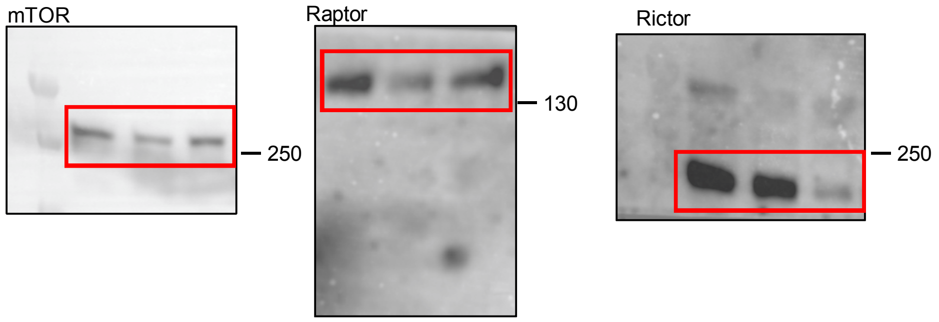

Supplementary Figure 12: Figure 9c uncropped western blots (CAF siRNA)

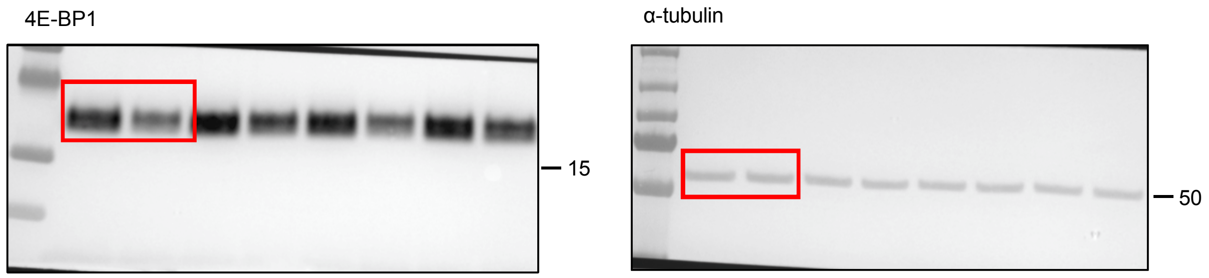

Supplementary Figure 12: Figure 9e uncropped western blots (HDF CRISPR)

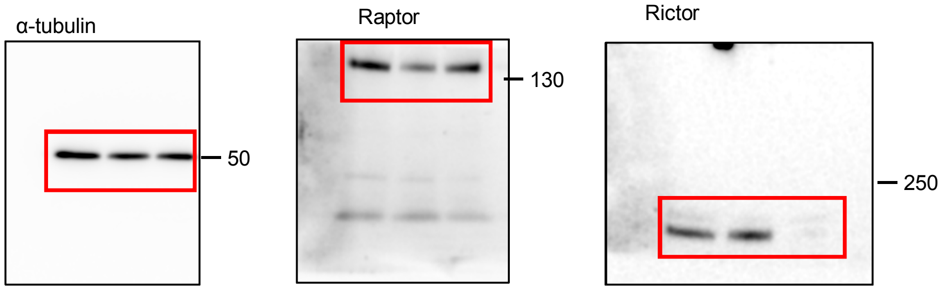

Supplementary Figure 12: Figure 9g uncropped western blots (HDF siRNA)

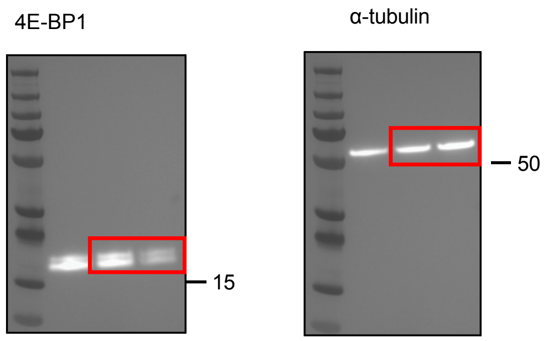

Supplementary Figure 12: Figure 9i uncropped western blots (HSC CRISPR)

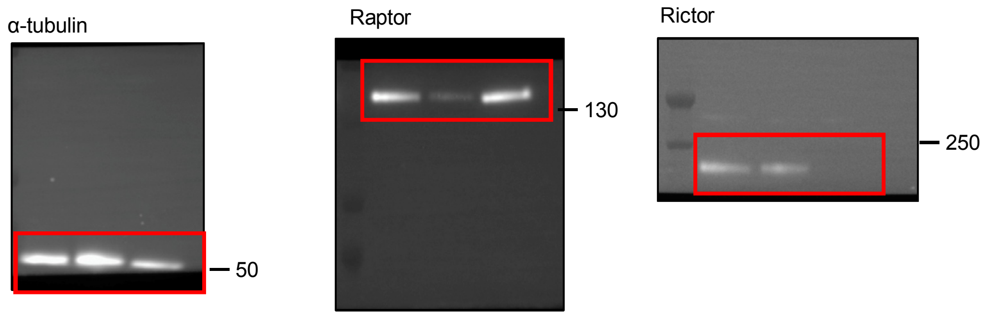

Supplementary Figure 12: Figure 9k uncropped western blots (HSC siRNA)

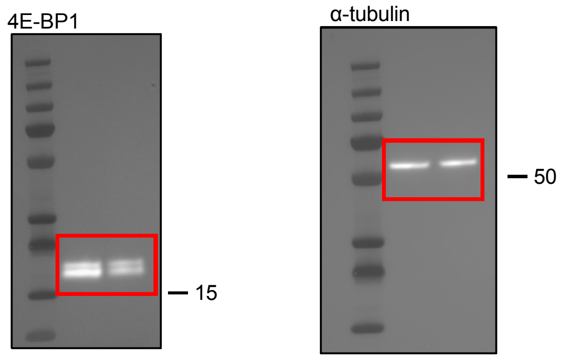

Supplementary Figure 13: Supplementary Figure 3a uncropped western blots (Torin-1)

p-SMAD2 (Ser465/467)

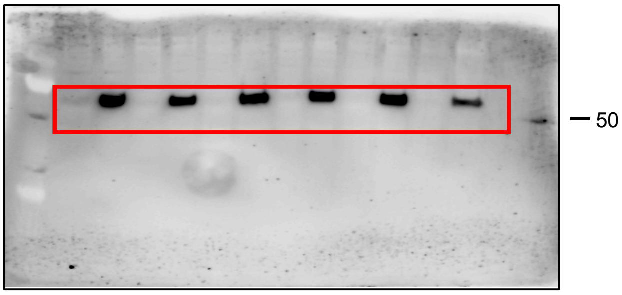

SMAD2

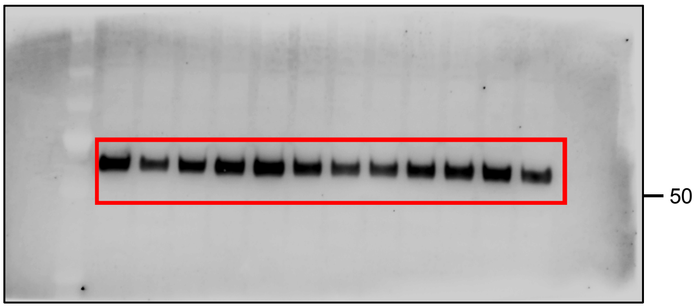

p-Akt (Thr308)

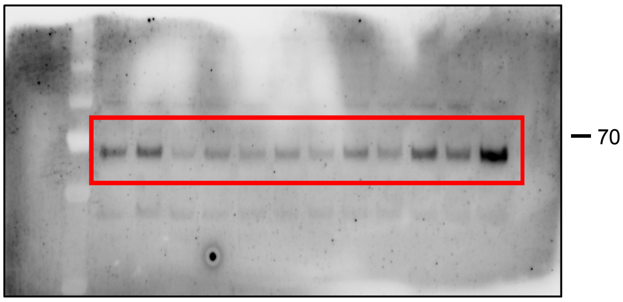

p-Akt (Ser473)

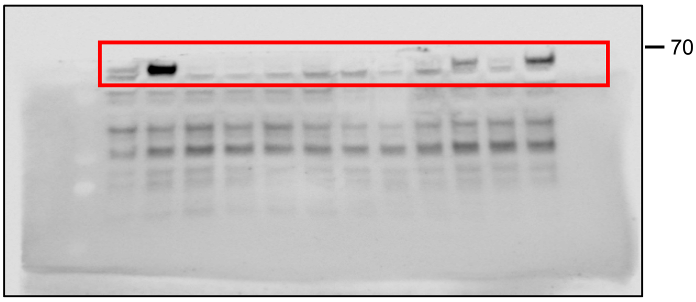

Akt

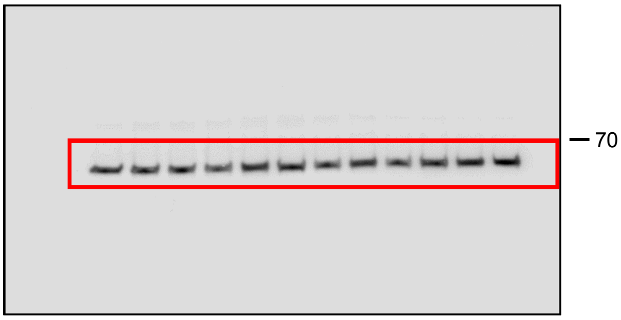

p-NDRG1 (Thr246)

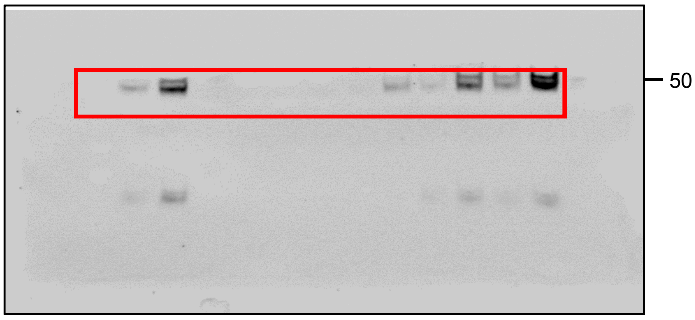

NDRG1

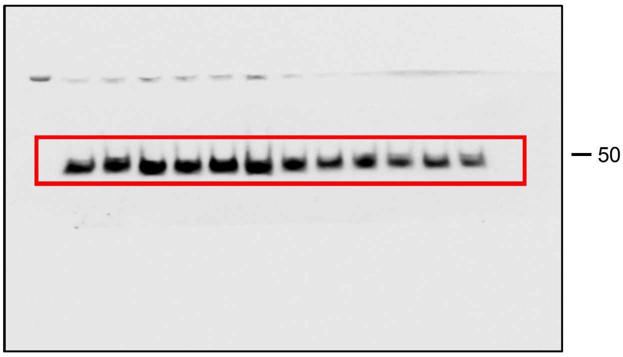

p-p70S6K (Thr389)

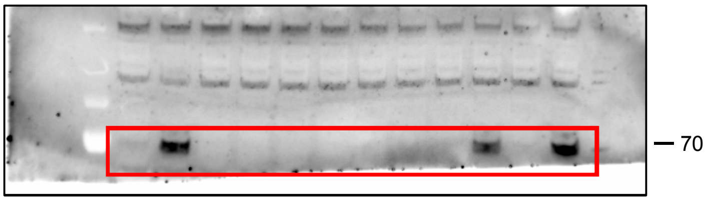

p70S6K

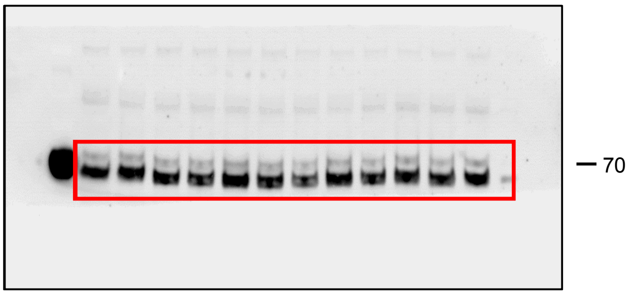

p-4E-BP1 (Ser65)

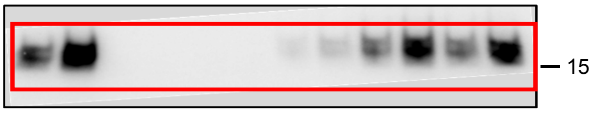

Supplementary Figure 13: Supplementary Figure 3a uncropped western blots (Torin-1) (Continued)

4E-BP1

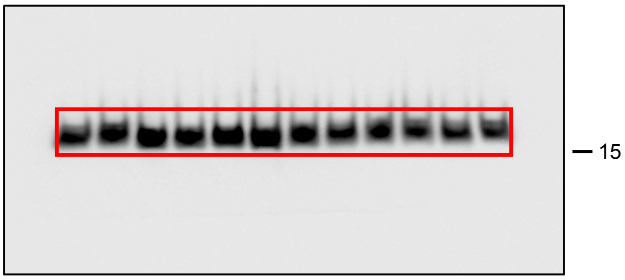

β actin

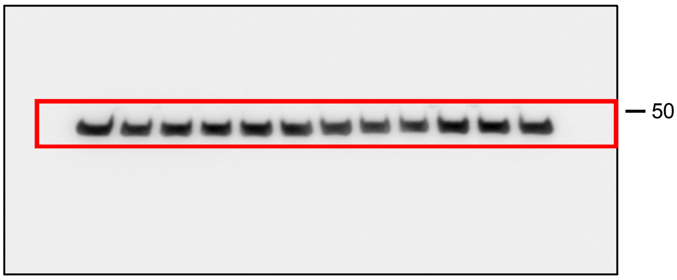

Supplementary Figure 13: Supplementary Figure 3b uncropped western blots (Compound 1)

p-SMAD2 (Ser465/467)

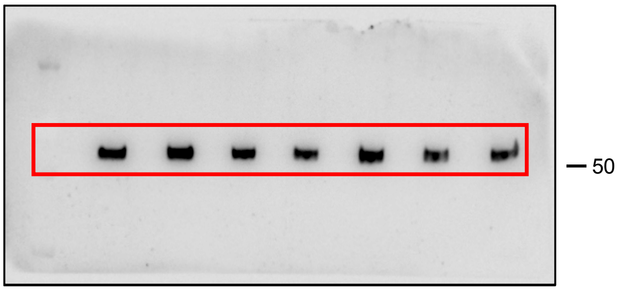

SMAD2

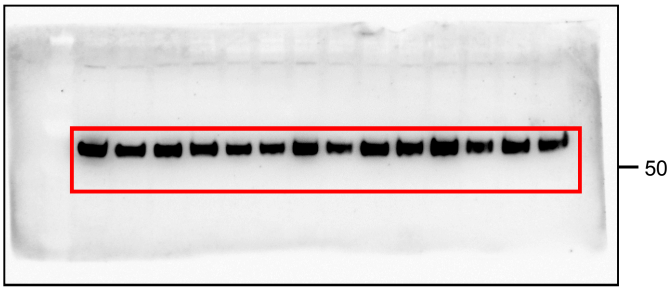

p-Akt (Thr308)

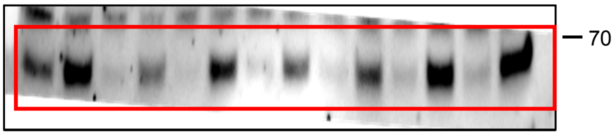

p-Akt (Ser473)

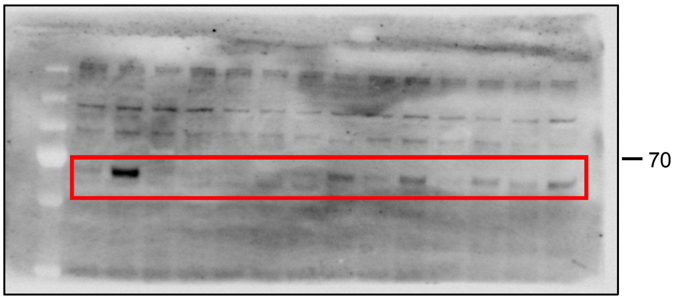

Akt

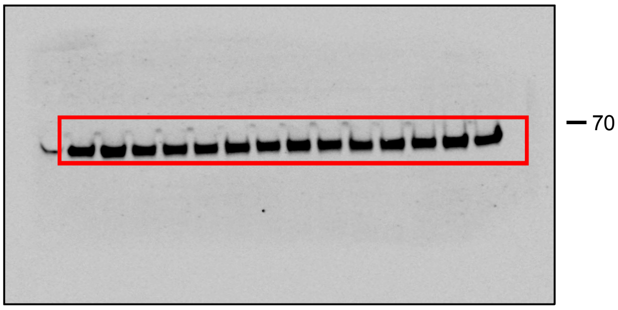

p-NDRG1 (Thr246)

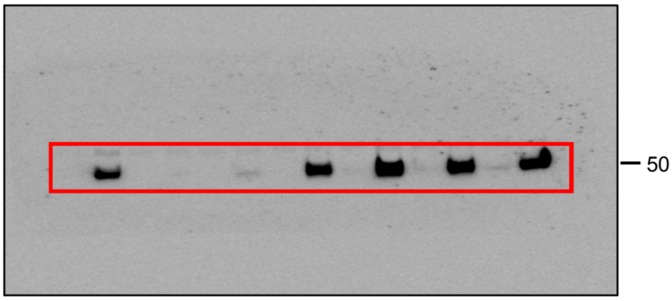

NDRG1

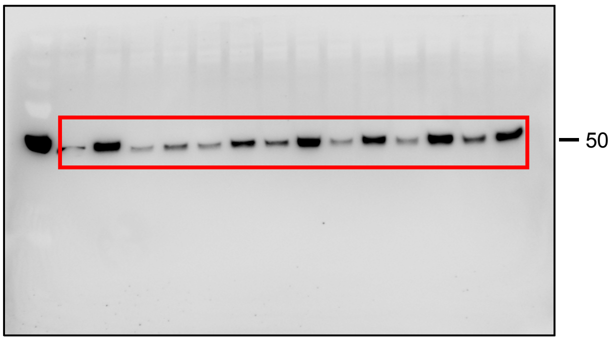

p-p70S6K (Thr389)

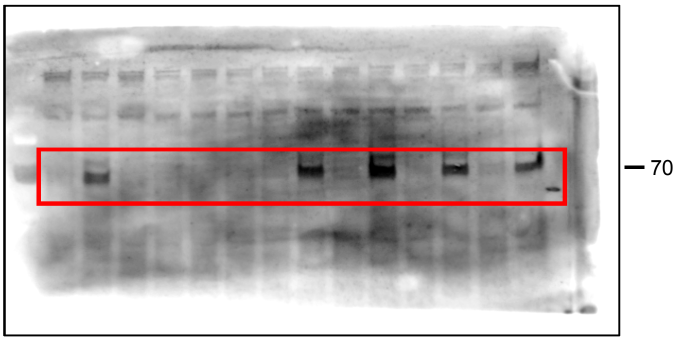

p70S6K

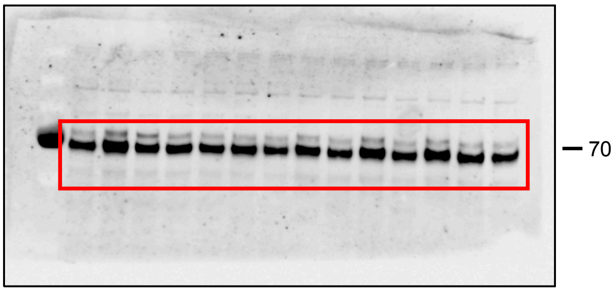

p-4E-BP1 (Ser65)

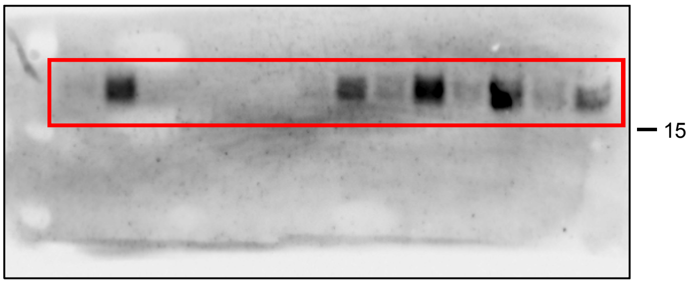

Supplementary Figure 13: Supplementary Figure 3b uncropped western blots (Compound 1) (Continued)

4E-BP1

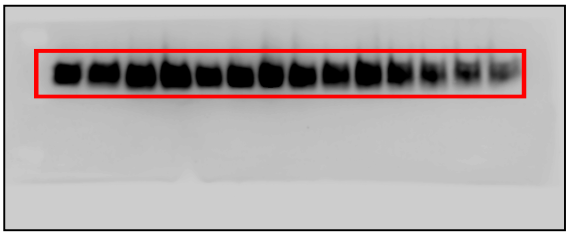

— 15

β actin

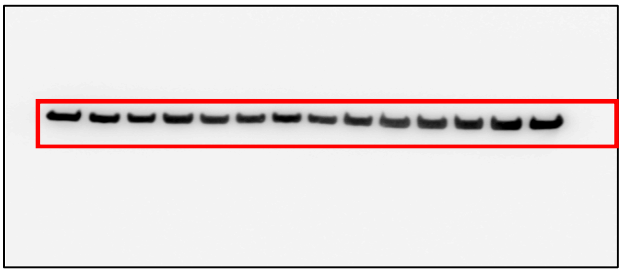

— 50

Supplementary Figure 14: Supplementary Figure 4a uncropped western blots

p-p70S6K (Thr389) (PI3K inhibitor)

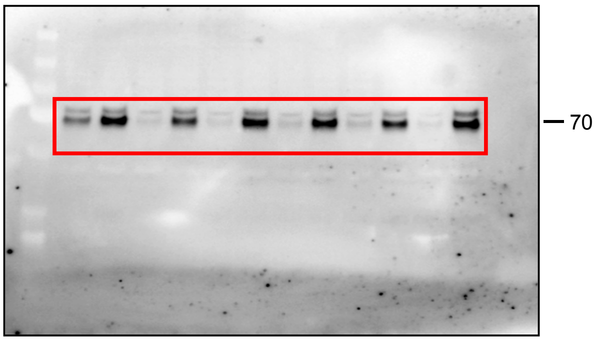

p70S6K (PI3K inhibitor)

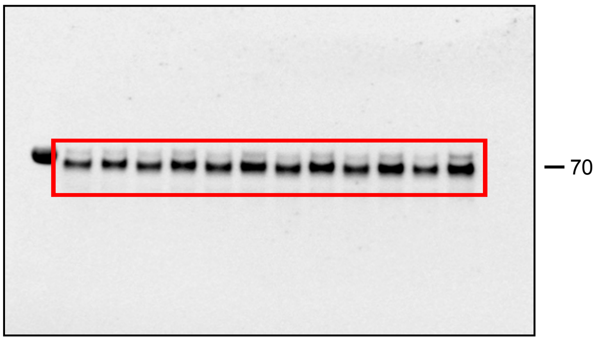

p-p70S6K (Thr389) (Akt inhibitor)

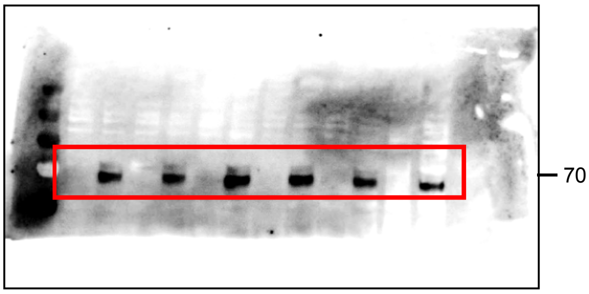

p-Akt (Ser473) (Akt inhibitor)

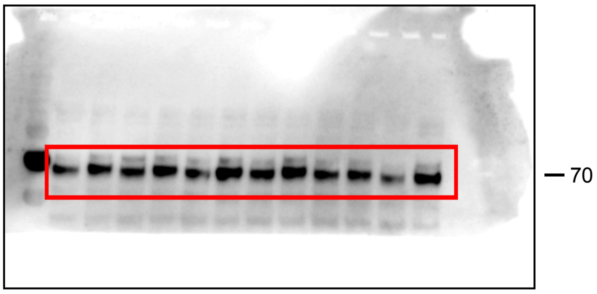

Supplementary Figure 14: Supplementary Figure 4b uncropped western blots

p-TSC2 S939 (Compound 2)

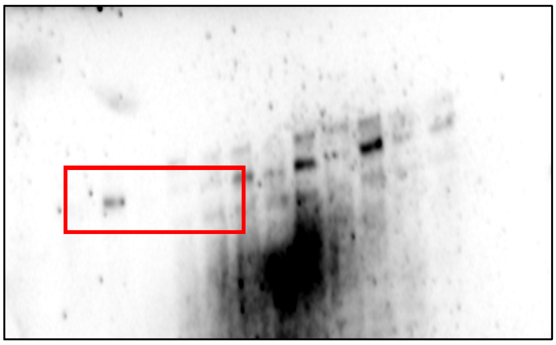

p-TSC2 S664 (Compound 2)

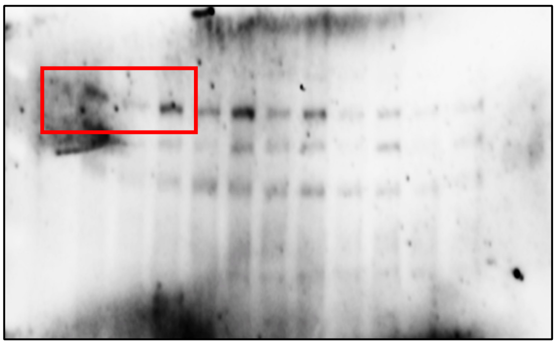

TSC2 (Compound 2)

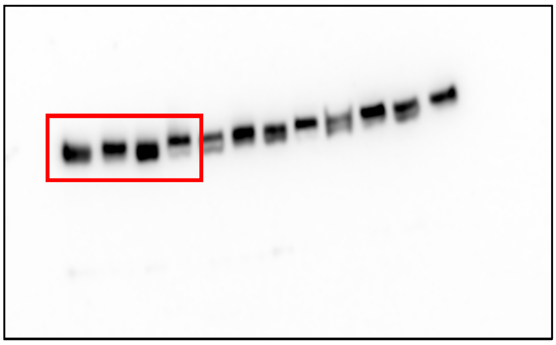

p-TSC2 S939 (MK2206)

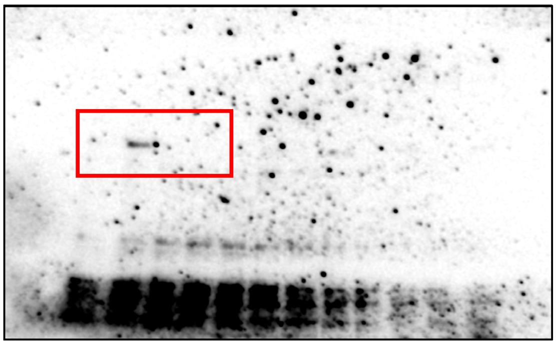

p-TSC2 S664 (MK2206)

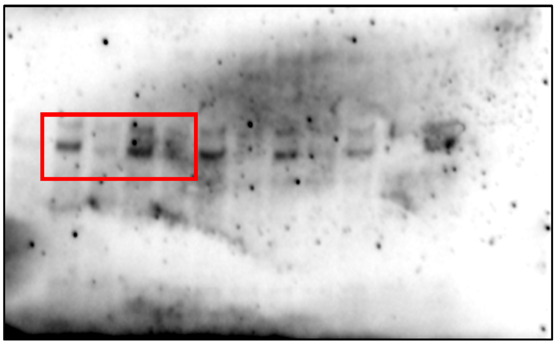

TSC2 (MK2206)

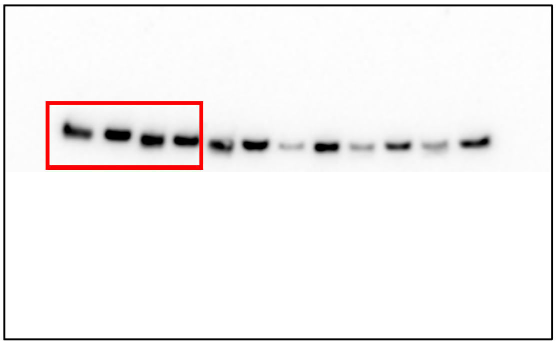

Supplementary Figure 14: Supplementary Figure 4b uncropped western blots (continued)

p-TSC2 S939 (GSK2334470)

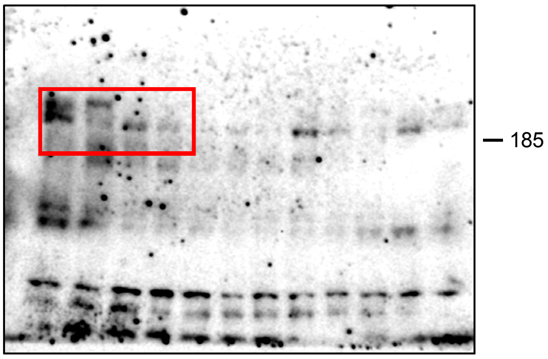

p-TSC2 S664 (GSK2334470)

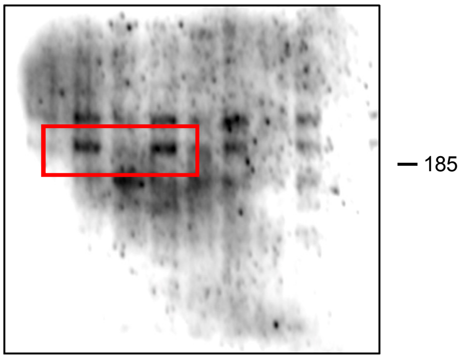

TSC2 (GSK2334470)

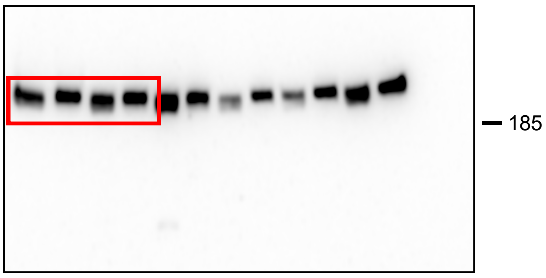

Supplementary Figure 15: Supplementary Figure 5a uncropped western blots

4E-BP1

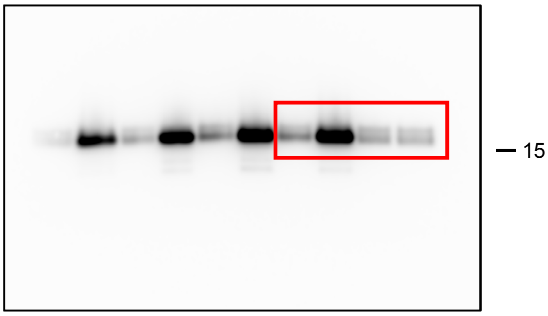

α tubulin

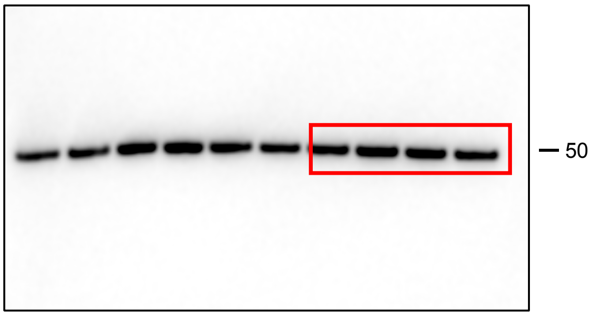

Supplement: Supplementary file 1 — Supplementary Information [file 41467_2018_7858_MOESM1_ESM.pdf]
