# Supplementary material for: The mTORC1/4E-BP1 axis represents a critical signaling node during fibrogenesis
Source: Nat Commun. 2019 Jan 2;10:6. doi: 10.1038/s41467-018-07858-8 (PMC6315032; doi:10.1038/s41467-018-07858-8)
Supplement: Supplementary file 2 — Description of Additional Supplementary Files [file 41467_2018_7858_MOESM2_ESM.docx]

**Description of Additional Supplementary Files**

**File Name**: Supplementary Data 1

**Description**: MS-based kinase binding profile of AZD8055 and CZ415 compounds across protein kinases identified from mixed human cell-line lysates. Data describe pIC50 and pKD app results from Kinobeads profiling. pKD app values are defined as the concentration of drug at which half-maximal competition of binding is observed, corrected by the influence of the immobilized ligand on the binding equilibrium using the Cheng-Prus off equation.

**File Name:** Supplementary Data 2.

**Description**: Expression proteomics data of IPF fibroblasts in response to TGF-β1, CZ415 and rapamycin. IPF human lung fibroblasts (IPF-HLFs) were pre-incubated with vehicle (0.1% DMSO), rapamycin (100nM) or the mTOR inhibitor CZ415 (5µM) prior to stimulation with TGF-β1 (1ng/ml) for 24 hours.
